# Crime against women in India: district-level risk estimation using the small area estimation approach

**DOI:** 10.3389/fpubh.2024.1362406

**Published:** 2024-07-16

**Authors:** B. S. Pooja, Vasudeva Guddattu, K. Aruna Rao

**Affiliations:** ^1^Department of Data Science, Prasanna School of Public Health, Manipal Academy of Higher Education, Manipal, India; ^2^Department of Statistics, Mangalore University, Mangalore, India

**Keywords:** women, crime, public health, small area estimation, spatial disparity, hotspots

## Abstract

**Background:**

The global prevalence of crimes against women has made it an enduring public health challenge that has persisted over time. The achievement of the 2030 Sustainable Development Goal (SDG) is intricately tied to the actions taken to prevent these crimes as their repercussions directly affect progress across various SDGs. This study aimed to provide a comprehensive examination of the prevalence of crimes against women across districts and states in India, analyzing changes from 2020 to 2022, and subsequently identifying associated factors.

**Methods:**

The study is an ecological analysis conducted across all districts of India using the data on crimes against women for the period 2020 and 2022 obtained from the National Crime Records Bureau (NCRB) of India. A small area estimation method was used to obtain district-level relative risks of crime against women for both periods. Hotspot analysis was carried out to identify the current hotspots and coldspots. Further spatial regression was used to identify the factors associated with crimes against women in the year 2022.

**Results:**

The results indicated a rise in the reported crime against women cases between 2020 and 2022. The rate of crimes against women at the national level was found to be 57 in the year 2020, whereas, in 2022, it increased to 67. The highest crime rate in the year 2022 was found to be 145 in Delhi, while Nagaland had the lowest crime rate of 5. The relative risk of crime against women varied from 0.046 to 4.68 in 2020, while in 2022, it spanned from 0.02 to 6.10. Significant hotspots were found in parts of Rajasthan, Madhya Pradesh, Haryana, Telangana, and Odisha. The results of the spatial error regression model showed that the sex ratio and the population density of the district have significant associations with the occurrence of crimes against women.

**Conclusion:**

The rise in the incidence of crime against women emphasizes the importance of tackling the spatial inequality in relative risk across Indian districts. By thoughtfully addressing this variation and conducting targeted studies in high-risk areas, we can enhance our understanding of the obstacles to implementing effective measures against violence targeting women.

## 1 Introduction

Violence against women, also referred to as crimes against women (CAW), encompasses any form of gender-based violence that causes or is likely to cause physical, sexual, or psychological harm or suffering to women. This includes threats of violence, coercion, or arbitrary deprivation of liberty, regardless of whether it occurs in public or private life (1). CAW is a pervasive issue worldwide, affecting women of diverse races, ethnicities, socioeconomic backgrounds, and nationalities. It is unfortunate to note that CAW has persisted throughout history as a longstanding concern. Despite being recognized as a punishable offense and numerous legislative efforts on a global scale, incidents of CAW continue to rise. The repercussions of CAW extend both at an individual level and on a global scale (2). For instance, on an individual level, it may lead to physical injury, mental health disorders, unintended pregnancies, or the spread of sexually transmitted infections, placing strain on the global healthcare system. Moreover, globally, CAW results in the violation of human rights, disparities in the legislative and judicial system, and reduced workforce engagement, among other consequences. Due to CAW intersecting with various aspects of social, economic, and gender equality, it can have an adverse effect on achieving the SDGs (2, 3). SDG-3 (good health and wellbeing), SDG-4 (quality education), SDG-5 (gender equality), SDG-8 (decent work and economic growth), SDG-10 (reduced inequality), SDG-11 (sustainable cities and communities), SDG-16 (peace, justice, and strong institutions), and SDG-17 (partnerships for the goals) are directly impacted by the consequences of CAW. Both on a national level, such as in India and worldwide, addressing CAW is imperative for attaining the SDGs by 2030 (3). According to the World Health Organization (WHO), one in every three women experiences at least one form of violence. It could be physical, emotional, or sexual violence either by intimate partner or non-partner violence (1). According to the United Nations Office on Drugs and Crime (UNODC), globally, approximately 89,000 women and girls were intentionally killed in 2022 (4). The Government of India has implemented numerous legislative and preventive measures aimed at safeguarding women and reducing incidents of CAW. These include the Criminal Law (Amendment) Act, 2018, the Legal Services Authorities (LSA) Act, the establishment of the National Commission for Women (NCW), initiatives such as Mission Shakti, Sakhi-One-stop Centres, along with campaigns such as Digital Shakti Campaign, and the Nirbhaya Fund (5). Despite these efforts, a recent annual report by the National Crime Records Bureau (NCRB) of India revealed a surge in the number of CAW by 15.3% in 2021 over 2020 and 4% in 2022 than the year 2021 (6). Fact sheets released by the National Family Health Survey (2019–21) revealed that the prevalence of domestic violence is 29.3% (7). Multiple studies across the world attempted to identify the underlying causes of CAW. Factors including women’s education status, poverty, alcohol and substance abuse, gender inequality, entrenched patriarchal norms, and cultural practices have all been implicated in contributing to CAW (3, 8–11). Despite observing faster economic growth, increased educational attainment, and greater female participation across various sectors, including education, space exploration, and decision-making, the country continues to grapple with a rising incidence of CAW (12, 13).

India, known for its vast diversity, consists of 28 states and 8 union territories (UTs), housing over 760 districts collectively. In India, districts serve as the fundamental units of local administration. For better planning of policies, preventive measures, and interventions to combat CAW, it is crucial to grasp the disparity in CAW both at the state and district levels, as well as to track how this dynamic is evolving. Though there are multiple studies related to crime in India, very few studies attempted to understand the risk of CAW among Indian districts (14–16). Thus, the study was undertaken to aid public health professionals, researchers, and policymakers in comprehending the changes in crime against women between 2020 and 2022, to estimate relative risks at the district level, and to identify the hotspots and coldspots among them. Furthermore, several kinds of literature have examined the different factors associated with the different types of CAW such as rape and domestic violence (3, 8–11). In the present study, we attempted to re-examine these associations for the variables for which we had recent data.

Given the absence of studies analyzing the relative risk of CAW at the district level across all Indian districts, our research fills this gap. Reliable district-level relative risk data for CAW obtained using the advanced statistical methodology for both the years 2020 and 2022 are highly beneficial in understanding the trends related to CAW at the district level, thereby improving decision-making and facilitating effective planning and program implementation.

## 2 Materials and methods

The data for this study were sourced from the National Crime Records Bureau (NCRB) of India, a government organization operating under the Ministry of Home Affairs. It serves as the nodal agency responsible for collecting, managing, and analyzing crime data at the national level. It publishes the facts and figures of different crimes at the national and state levels. Additionally, it provides district-level statistics of crimes under different headings such as district-wise Indian Penal Code (IPC) crimes, Special and Local Laws (SLL) crimes, CAW, and crimes against children for each year. Among those available datasets, we used the crime against women district-wise dataset for the years 2020 and 2022 which contains various forms of cognizable crimes committed against women (17). The dataset includes various forms of crime against women, such as cruelty by a husband or his relatives, assault with the intent to outrage a woman’s modesty, kidnapping and abduction, sexual violence against girl children, rape, dowry-related crimes, insults to a woman’s modesty, abetment of suicide, attempted rape, cybercrimes, immoral trafficking (women-centric only), domestic violence, human trafficking, miscarriage, murder with rape, acid attack, attempted acid attack, and the selling and buying of minor girls. This study considered the total CAW, including all instances specific to each Indian district that are cognizable (either under IPC or SLL). Information on female literacy, the proportion of males and females who drink alcohol, and the sex ratio was obtained from the district-level fact sheets published by the National Family Health Survey 2019–21 (18). Population projection for India and its states reported by the Government of India was used to obtain the district-level total female population and population density for the years 2020 and 2022 (19).

### 2.1 Methodology

The population projection report had information about the female population only for the states and not for the districts. Thus, the district-wise female population (FP) for the years 2020 and 2022 is calculated using the below-mentioned methodology.

Female population for the year 2020/2022:

Let $d_{ij}$ be the $i^{th}$ district in $j^{th}$ state and $S_{j}$ be the $j^{th}$ state. Population growth (PG)% for the $S_{j}$ from 2011 to 2020/2022 was obtained from the Population Projection Report India (2019). The total female base population for each district (for the year 2011) was obtained from the census 2011 report. Then


$$FP\;\mathrm{of}\;d_{ij}=\left(FP\;\mathrm{of}\;d_{ij}\;for\;2011\right)*\left(1+\frac{PG\%\mathrm{of}\;S_{j}\;}{100}\right)\text{.}$$

Similar to calculating the total female population for each of the districts, the population density was also calculated for the years 2020 and 2022 by using the 2011 population density data given by the Indian Census Bureau and the projected population density for each of the states obtained by Population Projection Report 2019 (19).

Since the female population data of the year 2011 was used as the base population to obtain the projected female population data of the districts for the years 2020 and 2022, adjustments were made to the district-level total number of CAW cases to account for the change in the number of districts from 2011 to 2020 and 2022. The total number of districts during the 2011 census was 640, and it increased to 741 in 2020 and 766 in 2022. Reliable data on the female population of the districts for the study period are key for the relative risk estimation. Because reliable data on the female population for the newly formed districts were unavailable, we decided to merge the CAW data of these districts with their parent districts. Hence, the total number of districts to which the relative risk of CAW is estimated is 640. The relative risk of CAW to the newly formed districts will be the same as that of the estimate obtained for its parent district.

For the years 2020 and 2022, CAW rates were calculated for each state and UTs of India and also for the whole nation as a number of CAW cases per 100,000 women. Crime risk estimates for each district could have been obtained by using the standardized incidence ratio (SIRs are direct estimates). The drawbacks of the SIRs are that they can be very unreliable, and spatial maps of the SIRs can badly distort the geographical distribution of the crime risks because the map tends to be dominated by areas of low population (20, 21). Hence, small area estimation (SAE) was used for estimating the relative risk of CAW for each Indian district for the years 2020 and 2022. SAE refers to a collection of statistical techniques used to obtain a precise estimate for the areas or domains to which the direct estimates are either imprecise or unreliable (20–22). Notably, districts are the small areas in the present study. Among the available SAE techniques, the spatial area-level SAE method is used to obtain the relative risk of CAW at the district level. This method gives a reliable estimate of the characteristics of interest for small areas by taking into account the spatial dependencies or relationships among neighboring areas. The spatial area-level SAE method incorporates spatial information into the estimation process, recognizing that nearby areas tend to exhibit similar characteristics or behaviors (20).

The general spatial area-level small area model can be described as follows (20, 21):

Let $O_{i}$ stands for the number of CAW observed in the $i^{th}$ district, = 1 …, *n*. Furthermore, $O_{i}\sim \mathrm{Poisson}\;\left(\mu_{i}=e_{i}r_{i}\right)$

where$e_{i}$ is the expected number of CAW in the $i^{th}$ district, and

$r_{i}$ is the relative risk of CAW associated with the $i^{th}$ district.

The relative risk of CAW can be modeled using the Besag–York–Mollie (BYM) model as follows: $\log\left(r_{i}\right)=\alpha +u_{i}+v_{i}\text{,}$ where α is the intercept that denotes the overall risk level, $u_{i}$ is the spatial structured random effect that models the spatial dependence between the relative risks, $v_{i}$ is an unstructured exchangeable random effect that models uncorrelated noise, and *u_i_* is modeled using conditional autoregressive (CAR) distribution. The neighborhood matrix was derived using a contiguity-based adjacency structure. $v_{i}$ is modeled using independent and identically distributed normal variables with zero mean and variance equal to $\sigma_{v}^{2}$ (21). The model was fitted using R 4.2.3 software, and the integrated nested Laplace approximation (INLA) technique was used to carry out approximate Bayesian inference (20, 21, 23). After estimating the $r_{i}$ for each district, districts were grouped into three categories. Districts with $r_{i}>1.1\;\mathrm{and}\;r_{i}<0.9$ are districts with high and low risk, respectively. Districts with *r_i_* between 0.9 and 1.1 are called as intermediate risk districts.

To generate the spatial map illustrating the estimated relative risks of CAW, we utilized QGIS 3.34.1 software. Moran’s I statistic was employed to evaluate the spatial autocorrelation. Moran’s I ranges from −1 to +1. A value close to +1 indicates strong positive spatial autocorrelation, indicating that areas with similar relative risk are in close proximity. Conversely, a value near −1 suggests strong negative spatial autocorrelation, implying that areas with dissimilar relative risk are clustered nearby. A value close to 0 indicates no spatial autocorrelation, indicating an absence of discernible patterns in the distribution of relative risk (24, 25). Following confirmation of spatial autocorrelation, the hotspot analysis was carried out to identify the significant areas where districts with a high relative risk of CAW were clustered together. These regions were termed as the hotspots of CAW. Conversely, ‘coldspots’ refer to significant areas where districts with low relative risk of CAW were clustered (25). GeoDa 1.22.0.4 software facilitated this hotspot analysis.

Among the variables that were found to have an association with the different types of CAW from existing literature, recent information was available for female literacy, sex ratio, population density, and % of men and women who drink alcohol corresponding to each district. Initially, both spatial lag and spatial error regression have been carried out to see how these variables are associated with the relative risks of CAW for the year 2022. Akaike’s information criterion (AIC) and coefficient of determination were used to identify the best-fitting model. A model with a lower AIC value and a model that explains larger variability of the outcome variable were considered as the better fitting model. Variables whose regression coefficient had a *p*-value of <0.05 were considered to have a significant association with CAW.

## 3 Results

### 3.1 Caw statistics at the national and state levels for the years 2020 and 2022

The results of the study revealed a notable difference in the occurrence of crimes between the years 2020 and 2022 both at the national and state levels. Specifically at the national level, in 2020, the total cognizable CAW was 371,503, whereas, in 2022, this number increased to 445,256.

Of the total cognizable CAW in the year 2020, the highest proportion (30%) was attributed to individuals close to the victim, including her husband or his relatives. Following closely, 23% of crimes were categorized as an assault on women with the intent to outrage their modesty. Additionally, nearly 17% of the total crimes took the form of kidnapping and abduction of women. Similar to the scenario in 2020, in 2022, the largest share of CAW, comprising 31.45%, was attributed to individuals closely associated with the victims. Moreover, nearly 19.16% of the total crimes consisted of kidnapping and abduction of women. Following closely behind, 18.71% of the crimes were categorized as an assault on women with the intent to outrage her modesty. A comprehensive breakdown of the distribution of various forms of crime at the national level is provided in Table 1.

**Table 1:** Various forms of crime against women and their contribution to the total incidents of crime against women for the years 2020 and 2022.

Type of crime	Total incidents for year 2020 (%)	Total incidents for year 2022 (%)
Cruelty by husband or his relatives	111,549 (30.03)	140,019 (31.45)
Assault on women with intent to outrage her modesty	85,392 (23.00)	83,344 (18.71)
Kidnapping and abduction of women	62,300 (16.80)	85,310 (19.16)
Protection of children from sexual violence act (girl child victims only)	46,123 (12.41)	62,095 (13.95)
Rape	28,046 (7.55)	31,516 (7.08)
Dowry-related (including dowry deaths)	17,332 (4.66)	19,929 (4.48)
Insult to the modesty of women	7,065 (1.90)	8,972 (2.02)
Abetment suicide of suicide	5,040 (1.36)	4,963 (1.11)
Attempted rape	3,741 (1.00)	3,288 (0.74)
Others	4,915 (1.32)	5,820 (1.31)
Total cognizable (IPC + SLL) crimes against women	371,503	445,256

Turning to the state-level statistics for both years as outlined in Table 2, it becomes apparent that in 2020, Uttar Pradesh (UP) reported the highest number of CAW (13.29%), followed by West Bengal (WB) with a contribution of 9.80% and Rajasthan with 9.30%. Maharashtra ranked fourth, accounting for 8.60% of reported cases after UP, WB, and Rajasthan. Notably, approximately 17 states and UTs accounted for less than 1% of the total cases of CAW for the year 2020. Uttar Pradesh maintained the highest total number of CAW incidents (65,743), with a slightly increased percentage of 14.77% in 2022. Following Uttar Pradesh, the states with the next highest proportions of CAW in 2022 were Maharashtra, accounting for 10.18%, and Rajasthan, with 10.12% of the total cases. All the states that contributed less than 1% to the total crime in 2020 remained consistent in 2022.

**Table 2:** Distribution of total crime against women across Indian states and UTs and crime rate corresponding to each state and UTs for the years 2020 and 2022.

	Year 2020		Year 2022	
States	Total CAW (%)	Crime rate (per 100,000 women)	Total CAW (%)	Crime rate (per 100,000 women)
Assam	26,352 (7.09)	155	14,148 (3.18)	81
Odisha	25,489 (6.86)	113	23,648 (5.31)	104
Delhi	10,093 (2.72)	107	14,247 (3.20)	145
Telangana	17,791 (4.79)	96	22,066 (4.96)	117
Haryana	13,000 (3.50)	95	16,743 (3.76)	119
Rajasthan	34,535 (9.30)	91	45,058 (10.12)	116
West Bengal	36,439 (9.81)	76	34,738 (7.80)	72
A&N Islands	143 (0.04)	76	178 (0.04)	94
Andhra Pradesh	17,089 (4.60)	65	25,503 (5.73)	96
Madhya Pradesh	25,640 (6.90)	64	32,765 (7.36)	79
Kerala	10,139 (2.73)	55	15,213 (3.42)	82
Chandigarh	301 (0.08)	55	325 (0.07)	58
Maharashtra	31,954 (8.60)	54	45,331 (10.18)	75
Jammu & Kashmir	3,405 (0.92)	54	3,716 (0.83)	58
Uttarakhand	2,846 (0.77)	52	4,337 (0.97)	77
Chhattisgarh	7,385 (1.99)	51	8,693 (1.95)	58
Uttar Pradesh	49,385 (13.29)	45	65,743 (14.77)	59
Himachal Pradesh	1,614 (0.43)	45	1,551 (0.35)	42
Lakshadweep	15 (0.004)	45	16 (0.00)	48
Tripura	874 (0.24)	44	752 (0.17)	37
Sikkim	140 (0.04)	44	179 (0.04)	55
Jharkhand	7,630 (2.05)	41	7,678 (1.72)	40
Karnataka	12,680 (3.41)	39	17,813 (4.00)	54
Arunachal Pradesh	281 (0.08)	38	335 (0.08)	45
Meghalaya	568 (0.15)	35	690 (0.15)	42
Punjab	4,838 (1.30)	34	5,572 (1.25)	38
Goa	219 (0.06)	29	273 (0.06)	35
Mizoram	172 (0.05)	29	147 (0.03)	24
Bihar	15,359 (4.13)	26	20,222 (4.96)	34
Gujarat	8,028 (2.16)	24	7,731 (1.74)	23
Tamil Nadu	6,630 (1.78)	17	9,207 (2.07)	24
D&N Haveli and Daman & Diu	61 (0.02)	17	126 (0.03)	31
Manipur	247 (0.07)	16	248 (0.06)	16
Puducherry	113 (0.03)	14	200 (0.04)	24
Ladakh	9 (0.002)	7	15 (0.00)	11
Nagaland	39 (0.01)	4	49 (0.01)	5

Although we understand the disparity in the occurrence of CAW across regions by just considering the total number of CAW cases, it is more meaningful to use the crime rate. The crime rate adjusts the incidence of crime to the total population residing in that area and gives us more meaningful and contextually relevant crime statistics to compare across the regions. CAW rate, which is calculated as crime cases per 100,000 women corresponding to each of the Indian states and UTs for the years 2020 and 2022, is given in Table 2. At the national level, the rate of CAW was found to be 57 CAW cases per 100,000 women for the year 2020. In 2022, this rate increased to 67 cases per 100,000 women.

**Figure 1:**
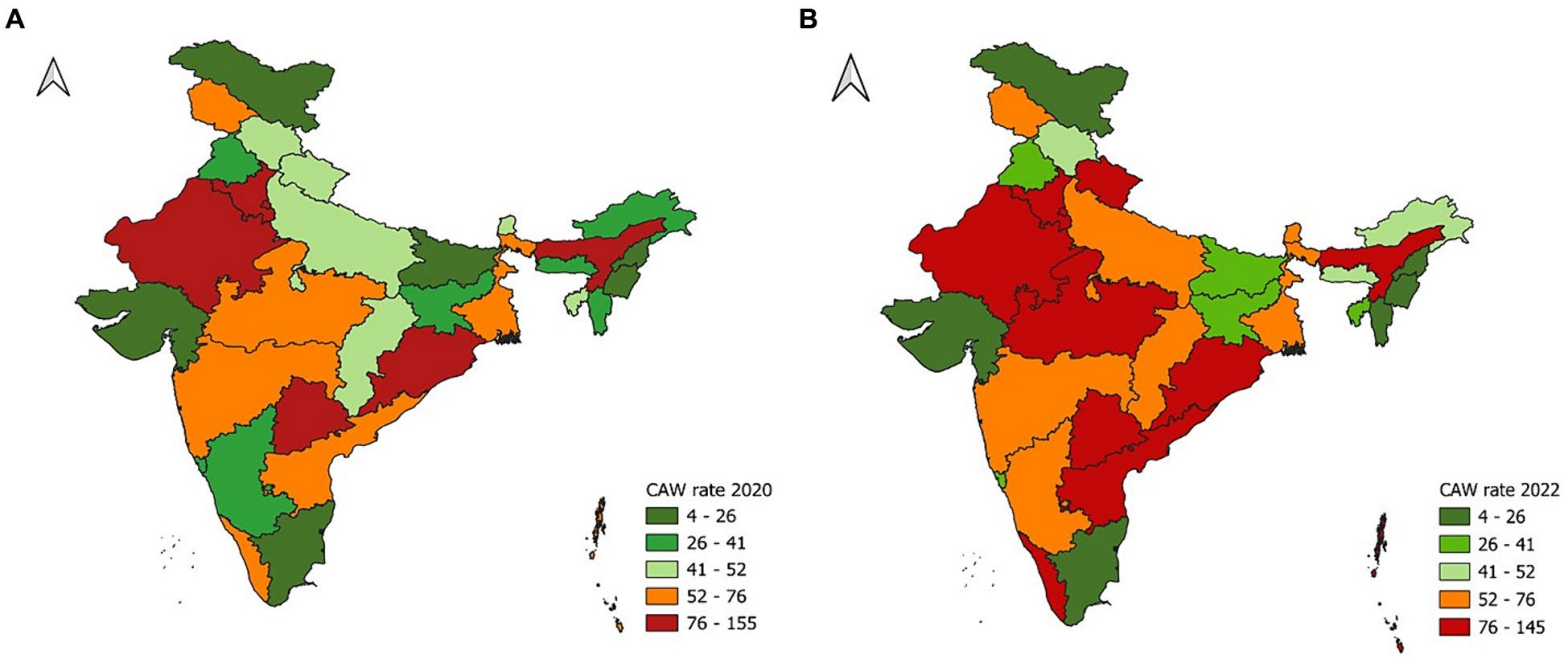
Spatial distribution of crime against women rate across Indian states and UTs for the years **(A)** 2020 and **(B)** 2022.

After adjusting for the female population corresponding to each state, it became evident that the rate of CAW varied significantly across Indian states in both years. In the year 2020, the crime rate varied from 4 to 155, while in 2022, it ranged from 5 to 145. In both years, the lowest crime rate was observed in Nagaland, followed by Ladakh, whereas Assam, which had the highest crime rate in the year 2020, was replaced by Delhi in the year 2022. Haryana, Telangana, Rajasthan, and Odisha continued to have the highest crime rates in both years. Notably, nine states and one UT exhibited CAW rates greater than the national average in 2020, while in 2022, it increased to twelve states and one UT. Among 36 states and UTs of India, 8 states (Assam, Odisha, West Bengal, Himachal Pradesh, Tripura, Jharkhand, Mizoram, and Gujarat) showed a decrease in CAW rates compared to 2020. Out of these eight states that showed a reduction in crime rate, a significant reduction was observed in the state of Assam (reduced by 74 cases). The remaining seven states had a reduction of less than 10 cases per 100,000 women. Except for Manipur, which showed no changes in the crime rate between 2020 and 2022, all the remaining 27 states and UTs of India showed an increase in the crime rate in 2022 compared to 2020. Within this group, Delhi witnessed the highest increase in crime rate by 38 cases, trailed by Andhra Pradesh with an increase of 31 cases, and subsequently by Kerala which saw an uptick of 27 cases. Rajasthan, Uttarakhand, and Haryana all experienced a similar increase in crime rate. In total, 12 states out of 27 states that exhibited an increase in crime rates saw a rise of less than 10 cases. The spatial distribution of crime rates across different Indian states and UTs for the years 2020 and 2022 is depicted in Figure 1.

### 3.2 Spatial distribution of CAW at the district level for the years 2020 and 2022

Similar to the disparities observed between states, variations in the distribution of CAW were evident among the districts within the states. Descriptive statistics of the characteristics considered in the study at the district level are given in Table 3.

**Table 3:** District-level descriptive statistics of the crime against women for the years 2020 and 2022 and other variables used in the study along with spatial error regression results.

Variables	Minimum	Maximum	Median	Mean	Standard deviation	Regression coefficients (*p*-value)
Total CAW (year 2022)	0	6,974	451	693	801.08	
Total CAW (year 2020)	0	6,976	388	581	676.86	
Crime rate (year 2022)	0	404	52	62	44.78	
Crime rate (year 2020)	0	266	44	55.22	41.83	
Relative risk (year 2022)	0.02	6.10	0.79	1.00	0.67	
Relative risk (year 2020)	0.05	4.68	0.78	0.98	0.73	
**Covariates used in the study**						
Women who drink alcohol (%)	0	42.8	0.5	2.58	5.72	−0.008 (0.30)
Men who drink alcohol (%)	0.1	68.4	19.4	22.25	12.47	0.001 **(0.76)**
Women who are literate (%)	37.1	99.7	74	73.24	12.74	0.002 (0.51)
Sex ratio (females per 1,000 males)	755	1,332	1,012	1020.6	73.9	−0.002 **(<0.01)**
Population density (the number of people per square kilometers)	1	45,151	419	1,092	3629.01	0.00001 (0.028)

In the year 2020, five districts reported zero incidents of CAW. These districts include Anjaw, Tawang, Tamenglong, Longleng, and Peren. Conversely, the district of North 24 Parganas in West Bengal recorded the highest number of CAW cases, totaling 6,976. Following closely, Rangareddy district in Andhra Pradesh reported 5,683 cases, while Mumbai Suburban in Maharashtra documented 4,583 cases. In contrast, in 2022, there were only three districts that reported no crime against women cases. Those districts are Jyotiba Phule Nagar, The Dangs, and Dibang Valley. Rangareddy districts of Andhra Pradesh have reported the highest number of CAW (6,974 cases), followed by Mumbai (6,293 cases) and Pune (5,434 cases) districts of Maharashtra.

District-level estimates of relative risks offer a more detailed perspective on CAW. These estimates, derived through small-area estimation methods, provide granular insights into the variations in CAW across all Indian districts. Complete details on the relative risk associated with each district are available in the Supplementary material for both years. Relative risks among the Indian districts in 2020 varied from 0.046 in the Ukhrul district of Manipur to 4.68 in the Dhubri district of Assam. Among the top 20 districts with the highest relative risks of CAW (relative risks greater than 3), 11 were from Assam (Dhubri, Barpeta, Kamrup Metropolitan, Morigaon, Darrang, Jorhat, Bongaigaon, Kamrup, Hailakandi, Nagaon, and Lakhimpur), 5 were from Odisha (Angul, Kendrapara, Jagatsinghapur, Dhenkanal, and Puri), 3 from Delhi (East Delhi, North Delhi, and New Delhi), and Rangareddy from Andhra Pradesh. In 2022, the relative risk of CAW across Indian districts ranged from 0.02 in Jyotiba Phule Nagar of Uttar Pradesh to 6.10 in the Mumbai district of Maharashtra. The top 20 high relative risks for CAW districts were from Delhi (North, Central, South, and New Delhi), Odisha (Debagarh, Angul, Kendrapara, and Malkangiri), Andhra Pradesh (Rangareddy, Warangal, and Krishna), Rajasthan (The Pratapgarh, Ajmer, and Bhilwara), Haryana (Panipat, Gurgaon, and Kurukshetra), Madhya Pradesh (Bhopal), and Nicobar.

**Figure 2:**
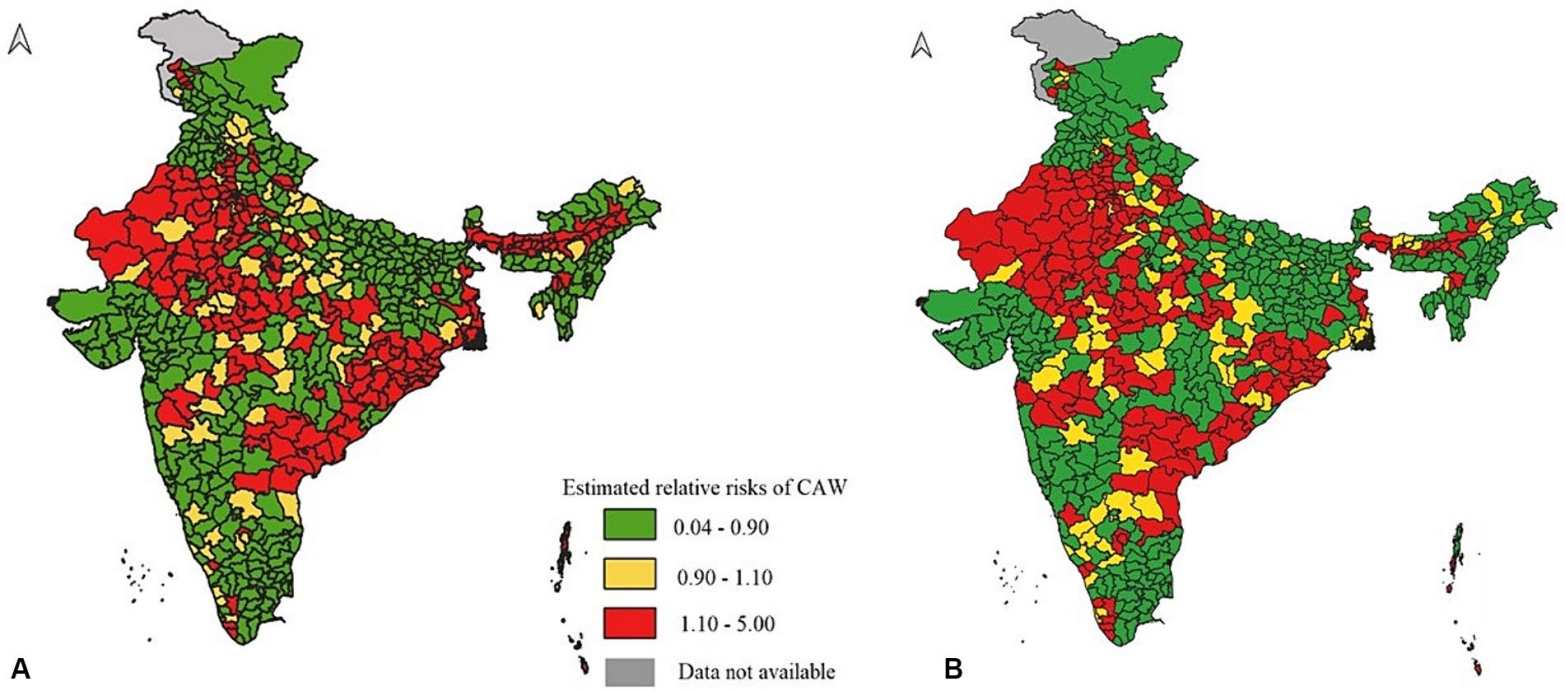
Spatial distribution of relative risks of crime against women across Indian districts for the years **(A)** 2020 and **(B)** 2022.

**Figure 3:**
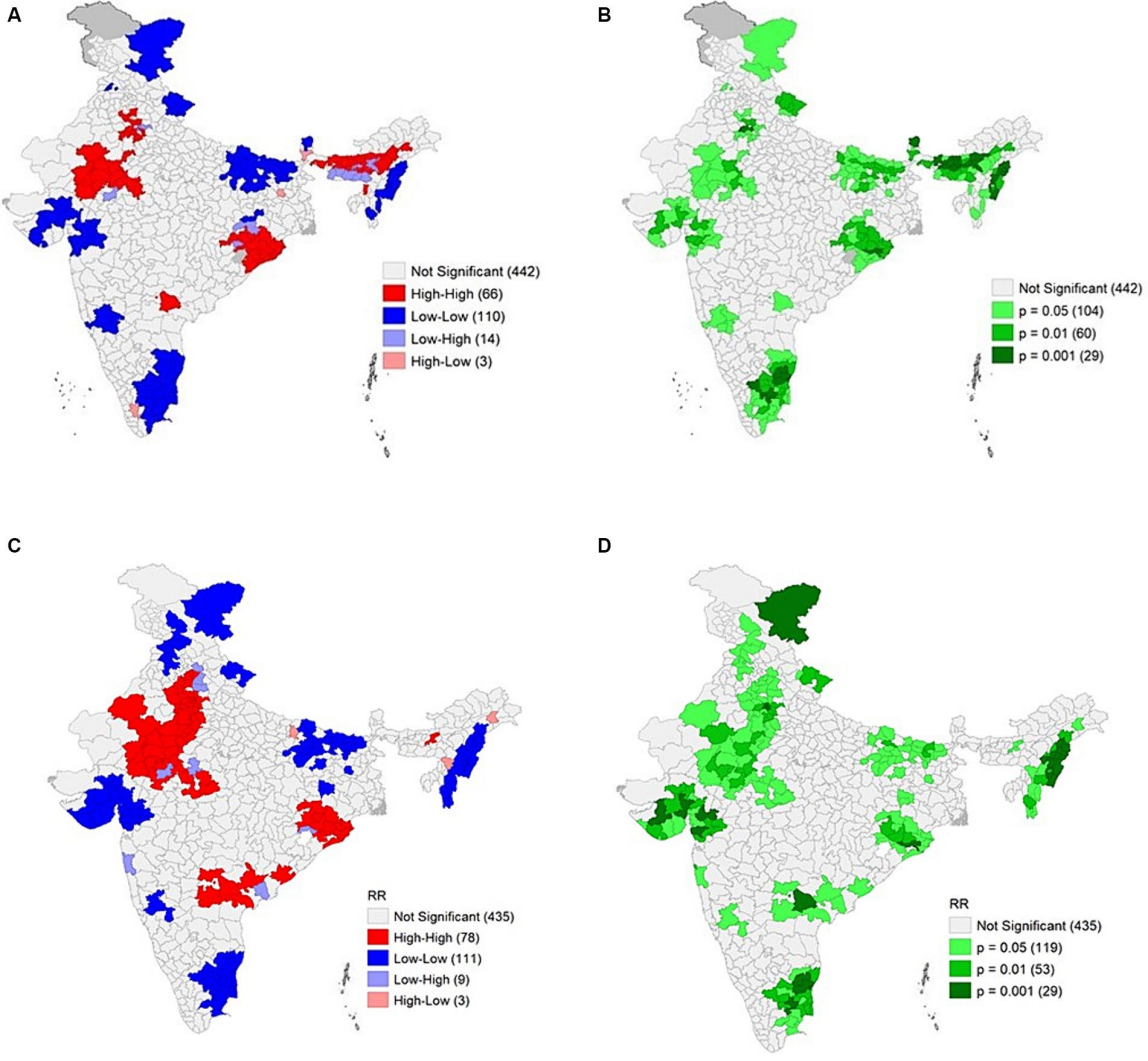
The results of hotspot analysis for the years 2020 and 2022: **(A)** Cluster map of crime against women relative risk distribution and corresponding **(B)** significance map for the year 2020, **(C)** Cluster map of crime against women relative risk distribution and corresponding **(D)** significance map for the year 2022.

In both years, approximately 57% of the total districts had a low relative risk for CAW, while 12% had an intermediate risk and 31% of the total districts had a high relative risk for CAW. To better understand the trend in the spatial distribution of the relative risk of CAW in each district, spatial maps have been produced for both years and are presented in Figure 2. By closely comparing the spatial maps of the years 2020 and 2022, the following conclusions were drawn: the number of districts with high relative risk in Assam has decreased over the period, and some districts of Maharashtra, Rajasthan, Madhya Pradesh, Andhra Pradesh, Telangana, and Odisha, which were either low or intermediate risk regions turned into high-risk regions. Overall, from Figure 2, there is clear evidence that there is a shift in the spatial distribution of relative risk of CAW over time. To confirm the significance of these high-risk and low-risk areas that we obtained during both years, hotspot analysis was carried out, and the results are presented in Figure 3. Moran’s I for the spatial distribution of relative risks at the district level was found to be 0.54 and 0.44 for the years 2020 and 2022, respectively, indicating the presence of spatial clustering of Indian districts concerning the relative risk of CAW. A significant change in the location, size, and significance of the hotspots has been observed between the cluster map corresponding to the years 2020 (Figure 3A) and 2022 (Figure 3C) and their corresponding significance maps (Figures 3B,D). The major and significant hotspots located in Assam in the year 2020 have decreased both in size and significance. The hotspot that was present in the year 2020 in the parts of Rajasthan, Madhya Pradesh, and Haryana has increased both in cluster size and significance. The new major and significant hotspot was identified in the year 2022 in the parts of Telangana and Andhra Pradesh. Contrary to these findings, minimal change was observed in the hotspots detected in parts of Odisha when comparing the years 2020 and 2022. Table 4 provides a detailed list of districts belonging to hotspots and coldspots for the year 2022.

**Table 4:** List of districts corresponding to hotspots and coldspots for relative risk of crime against women for the year 2022.

Hotspots (high-high regions)	**Cluster 1**: Ambala, Bhiwani, Faridabad, Gurgaon, Hisar, Jhajjar, Kaithal, Karnal, Kurukshetra, Mewat, Palwal, Rewari, Rohtak, Sonipat, Yamunanagar, Jind, Neemuch, Raisen, Rajgarh, Ratlam, Sehore, Vidisha, Central Delhi, East Delhi, New Delhi, North Delhi, North East Delhi, North West Delhi, South Delhi, South West Delhi, West Delhi, Ajmer, Bharatpur, Bhilwara, Bikaner, Bundi, Chittaurgarh, Churu, Hanumangarh, Jaipur, Jhalawar, Kota, Nagaur, Pali, Rajsamand, Tonk, Udaipur, Dausa, Karauli, Sawai Madhopur, Baghpat, Gautam Buddha Nagar, and Mathura; **Cluster 2:** Angul, Bhadrak, Cuttack, Debagarh, Dhenkanal, Jagatsinghapur, Jajapur, Kendrapara, Kendujhar, Nayagarh, Puri, Sambalpur, and Sundargarh; **Cluster 3:** East Godavari, Guntur, Hyderabad, Khammam, Mahbubnagar, Medak, Nalgonda, and Visakhapatnam; and **Cluster 4:** Darrang, Kamrup Metropolitan
Coldspots (low-low regions)	**Cluster 1:** Ariyalur, Chennai, Cuddalore, Dharmapuri, Erode, Kancheepuram, Karur, Madurai, Namakkal, Perambalur, Pudukkottai, Ramanathapuram, Salem, Sivaganga, Thanjavur, Tiruchirappalli, Tiruvannamalai, Vellore, Viluppuram, and Nagapattinam; **Cluster 2:** Ahmadabad, Amreli, Anand, Bharuch, Bhavnagar, Gandhinagar, Junagadh, Kachchh, Kheda, Mahesana, Narmada, Navsari, Patan, Porbandar, Rajkot, Surat, Surendranagar, Vadodara, and Tapi; **Cluster 3:** Araria, Bhojpur, Buxar, Darbhanga, Gaya, Gopalganj, Kaimur (bhabua), Khagaria, Madhepura, Madhubani, Munger, Muzaffarpur, Pashchim Champaran, Patna, Purnia, Rohtas, Saharsa, Samastipur, Saran (chhapra), Sitamarhi, Siwan, Supaul, Deoghar, Giridih, Khunti, Palamu, Ranchi, and Belgaum; **Cluster 4:** Bishnupur, Chandel, Churachandpur, Imphal East, Senapati, Tamenglong, Thoubal, Ukhrul, Aizawl, Champhai, Lawngtlai, Lunglei, Saiha, Serchhip, Dimapur, Kiphire, Kohima, Longleng, Mokokchung, Mon, Peren, Phek, Tuensang, Wokha, and Zunheboto; and **Cluster 5:** Barnala, Faridkot, Gurdaspur, Hoshiarpur, Jalandhar, Kapurthala, and Ludhiana.

The spatial lag model yielded the Akaike information criterion (AIC) of 1,078 with a coefficient of determination $\left(R^{2}\right)$ of 34%. In comparison, the spatial error model resulted in an AIC of 1,071 with an $R^{2}$ of 35.67%. Since the spatial error model had the lowest AIC and high *R*^2^, it was identified as the better model. The results of the spatial error regression model are presented in Table 3. Among the five variables considered, the sex ratio and population density had a significant association with the relative risks of CAW, whereas the other three variables, such as female literacy and the percentage of men and women who drink alcohol, had no significant association with the relative risks of CAW.

## 4 Discussion

In this ecological study, our primary aim was to examine the trends in the distribution of CAW across India’s states and territories. Our objectives extended to estimating the relative risk of CAW among the Indian districts, identifying the hotspots of CAW, and discerning the variables associated with this risk. The findings of our study evidently revealed an increase in crime against women over the study period. In both years, the contribution of crimes classified as cruelty by a husband or his family to the total crime against women remains the same and high. This type of crime is also termed domestic violence. These statistics corroborate reports published by the NFHS-5 regarding the prevalence of domestic violence in India (7). Despite governmental measures such as the Protection of Women from Domestic Violence (PWDV) Act of 2005, the National Policy for Women (2016), gender budgeting in all government interventions, and a One-Stop Centers scheme to support the victims (26, 27), the persistence of domestic violence remains a pressing concern. There are numerous studies conducted, both in India and globally, to understand domestic violence through its different dimensions. Various kinds of literature studies are available to identify the causes or factors that influence the prevalence of domestic violence at the individual level, family level, and community level (28–30). These studies can be utilized for designing new policies or programs or to design operational frameworks to combat domestic violence, which further will help in combating the crime against women.

Generally, there was an increase in the total number of CAW incidents and crime rates across most states from 2020 to 2022. However, there were exceptions, such as Assam, Odisha, Himachal Pradesh, Tripura, Mizoram, and Gujarat, which experienced a decline in both total CAW cases and crime rates. This apparent increase in CAW could be attributed to several factors. Some of the potential reasons could be population size, improved reporting practices, and law enforcement practices. Given that Uttar Pradesh, Maharashtra, and West Bengal rank among the top five most populous states in India (19), even if their crime rates were relatively low, the absolute number of reported cases of CAW cases may still be high. Improved reporting and law enforcement practices have likely empowered victims and their families to come forward and report crimes more frequently (31). Additionally, the increase in crime rates among Indian states could stem from socioeconomic factors and the fulfillment or lack of people’s needs (31). The reason that significant decreases in total CAW cases and crime rates (such as Assam) could be due to enhanced crime prevention measures and better investigations leading to an arrest and charge sheeting of the accused (31, 32). Revisiting the programs and policy implementation taken up to prevent CAW would help in reducing the increasing trend of CAW. Furthermore, the states that have the lower crime rate and those states that showed a decrease in the overall CAW cases and crime rate may contain some regions that have high risks of CAW. To understand the disparity in the distribution of CAW cases and the corresponding relative risks, a district-level analysis performed in the present study will be highly useful. Districts such as Mumbai (Maharashtra), North Delhi, and Debagarh (Odisha) had far higher CAW rates compared to their parent state. This within-state variability has to be considered in formulating interventions and making decisions. A positive value of Moran’s I indicates that the relative risk of CAW is related to the neighboring regions. The disparity in the count of districts exhibiting high or low relative risks of CAW compared to the number of districts designated as hotspots and coldspots suggests that not all districts identified with high or low relative risks authentically represent areas of heightened or reduced relative risk. These instances may stem from the random distribution of the CAW phenomenon. The list of significant hotspots and coldspots obtained in this study serves as the list of priority districts for the concerned individuals, agencies, and ministries working on preventing the CAW. Significant hotspots for the relative risk of crime against women found in the present study are majorly situated in the states of Andhra Pradesh, Telangana, Haryana, Madhya Pradesh, NCT of Delhi, Odisha, Rajasthan, and Uttar Pradesh. These are the priority states that need quick attention.

The association between the sex ratio, population density, and the relative risk of CAW is in line with previous studies (8). The sex ratio is one of the indicators that measures gender equality. This indicates that as the sex ratio increases (more women relative to men), it may potentially lead to a decrease in certain types of CAW (33, 34). For example, in societies, where women are in the numerical majority, they may have more bargaining power either in relationships or marriages. This increased bargaining power could lead to greater autonomy and decision-making power for women, which, in turn, could reduce their vulnerability to certain types of crimes, such as domestic violence or marital rape (34). The reasons for not observing the significant association between female literacy and men or women who drink alcohol could be attributed to a variety of factors. Since we worked with the aggregated data in the present study, it might have masked the individual-level relationships and introduced the bias (35). Additionally, the reason for no significant association between relative risks of crime against women and women’s literacy could be attributed to the widespread achievement of higher education across almost all regions (7). To tackle the issues arising due to the aggregated nature of the data analyzed, we recommend taking up individual-level exploratory studies in high-priority areas, which can further help the researchers and public health professionals involved in combating CAW.

The limitations of existing district-level CAW studies lie in their focus on specific forms of crime, such as rape incidence and dowry deaths, either across all districts of India or within individual states. It is crucial to recognize that all forms of CAW are significant and should not be overlooked or considered less serious. Every act of violence irrespective of its magnitude or severity demands attention, recognition, and a concerted effort to address and prevent it. Our study stands out for its comprehensive approach, considering all potential forms of violence across all states, UTs, and their corresponding districts. Furthermore, the comparative nature of our study, which assessed the relative risk of CAW for both 2020 and 2022, addresses a gap in the existing literature.

### 4.1 Limitations of the study

The results presented in this study have to be considered with the following limitations:

1. The study used data from the National Crime Records Bureau to present data on CAW, which were reported to the official agency. The number of crimes reported to official agencies is much less than the actual crimes taking place within India. Hence, underreporting of the data is one of the limitations of this study which is not unique to this study but to all the crime-related studies that use the data published from NCRB.
2. Lack of information on additional socio-economic variables was found to have an association with CAW in previous studies.

Despite the acknowledged limitations, the study stands strong by providing the current distribution of relative risks of CAW across Indian districts and the list of districts that need quick attention and strong interventions.

## 5 Conclusion

Although several measures have been taken up by the Government of India to combat the crime against women, the crime rate is still increasing, which is quite alarming. The consequences of CAW extend beyond individuals and encompass the wider community and society to which these women belong. Probable challenges that impede the prevention of CAW across the nation could be deep-rooted societal norms, persisting economic disparities, lack of empowerment, insufficient legal awareness, and inadequate implementation of laws. CAW is a complex issue, and therefore, one particular approach will not fit all the regions. Hence, region-specific interventions considering socio-cultural diversity within the region and demographic differences between the regions will be more effective in reducing the CAW in the regions.

## Data availability statement

Publicly available datasets were analyzed in this study. This data can be found here: crime against women district wise data for the year 2020 can be obtained from National Crime Record Bureau (NCRB) India website (17). Additional variables used in the study can be obtained from Census Bureau of India (https://censusindia.gov.in/census.website/) (19) and NFHS-5 (18).

## Author contributions

BP: Conceptualization, Data curation, Formal analysis, Methodology, Writing – original draft, Writing – review & editing. VG: Conceptualization, Methodology, Resources, Supervision, Validation, Visualization, Writing – review & editing. KR: Conceptualization, Methodology, Writing – review & editing.
