# Supplementary material for: Crime against women in India: district-level risk estimation using the small area estimation approach
Source: Front Public Health. 2024 Jul 16;12:1362406. doi: 10.3389/fpubh.2024.1362406 (PMC11288248; doi:10.3389/fpubh.2024.1362406)
Supplement: Supplementary file 1 [file Table_1.DOCX]

Supplementary Material

# Supplementary Data:

# Table containing the estimated relative risk (RR) of Indian districts obtained from small area estimation method for the year 2020 and 2022 (RR- Estimated relative risk, LL- Lower Limit of RR, UL- Upper Limit of RR)

|  | | | For the year 2020 | | | For the year 2022 | | |
| --- | --- | --- | --- | --- | --- | --- | --- | --- |
| DISTRICT | state name | censuscode | RR | LL | UL | RR | LL | UL |
| Kupwara | Jammu & Kashmir | 1 | 1.13 | 1.00 | 1.26 | 0.78 | 0.69 | 0.89 |
| Badgam | Jammu & Kashmir | 2 | 1.52 | 1.36 | 1.68 | 1.06 | 0.95 | 1.19 |
| Leh (ladakh) | Jammu & Kashmir | 3 | 0.20 | 0.09 | 0.37 | 0.23 | 0.12 | 0.40 |
| Kargil | Jammu & Kashmir | 4 | 0.20 | 0.11 | 0.33 | 0.25 | 0.14 | 0.39 |
| Punch | Jammu & Kashmir | 5 | 0.90 | 0.75 | 1.06 | 0.81 | 0.68 | 0.95 |
| Rajouri | Jammu & Kashmir | 6 | 1.00 | 0.86 | 1.15 | 1.40 | 1.25 | 1.56 |
| Kathua | Jammu & Kashmir | 7 | 0.69 | 0.58 | 0.82 | 0.44 | 0.36 | 0.53 |
| Baramula | Jammu & Kashmir | 8 | 1.32 | 1.19 | 1.45 | 0.61 | 0.53 | 0.69 |
| Bandipore | Jammu & Kashmir | 9 | 0.62 | 0.48 | 0.76 | 1.33 | 1.15 | 1.54 |
| Srinagar | Jammu & Kashmir | 10 | 0.81 | 0.72 | 0.91 | 1.07 | 0.97 | 1.17 |
| Ganderbal | Jammu & Kashmir | 11 | 1.35 | 1.12 | 1.61 | 1.60 | 1.37 | 1.85 |
| Pulwama | Jammu & Kashmir | 12 | 0.61 | 0.50 | 0.73 | 1.09 | 0.95 | 1.24 |
| Shupiyan | Jammu & Kashmir | 13 | 1.87 | 1.59 | 2.18 | 0.87 | 0.70 | 1.07 |
| Anantnag | Jammu & Kashmir | 14 | 0.79 | 0.70 | 0.89 | 0.61 | 0.54 | 0.69 |
| Kulgam | Jammu & Kashmir | 15 | 2.08 | 1.84 | 2.33 | 1.30 | 1.13 | 1.49 |
| Doda | Jammu & Kashmir | 16 | 0.68 | 0.54 | 0.83 | 0.74 | 0.61 | 0.89 |
| Ramban | Jammu & Kashmir | 17 | 0.77 | 0.59 | 0.96 | 0.66 | 0.51 | 0.83 |
| Kishtwar | Jammu & Kashmir | 18 | 0.58 | 0.42 | 0.76 | 0.52 | 0.38 | 0.69 |
| Udhampur | Jammu & Kashmir | 19 | 0.69 | 0.57 | 0.82 | 0.76 | 0.64 | 0.89 |
| Reasi | Jammu & Kashmir | 20 | 0.58 | 0.44 | 0.74 | 0.79 | 0.64 | 0.96 |
| Jammu | Jammu & Kashmir | 21 | 0.71 | 0.63 | 0.79 | 0.83 | 0.76 | 0.91 |
| Samba | Jammu & Kashmir | 22 | 0.64 | 0.49 | 0.81 | 0.43 | 0.32 | 0.56 |
| Chamba | Himachal Pradesh | 23 | 0.62 | 0.50 | 0.75 | 0.55 | 0.45 | 0.66 |
| Kangra | Himachal Pradesh | 24 | 0.57 | 0.50 | 0.64 | 0.52 | 0.46 | 0.58 |
| Lahul & Spiti | Himachal Pradesh | 25 | 0.39 | 0.18 | 0.70 | 0.41 | 0.20 | 0.74 |
| Kullu | Himachal Pradesh | 26 | 1.01 | 0.84 | 1.19 | 0.73 | 0.60 | 0.87 |
| Mandi | Himachal Pradesh | 27 | 0.91 | 0.81 | 1.02 | 0.63 | 0.55 | 0.71 |
| Hamirpur | Himachal Pradesh | 28 | 0.60 | 0.49 | 0.73 | 0.58 | 0.48 | 0.71 |
| Una | Himachal Pradesh | 29 | 0.62 | 0.50 | 0.74 | 0.63 | 0.52 | 0.75 |
| Bilaspur | Himachal Pradesh | 30 | 1.04 | 0.86 | 1.23 | 0.69 | 0.57 | 0.84 |
| Solan | Himachal Pradesh | 31 | 0.95 | 0.81 | 1.10 | 0.91 | 0.78 | 1.05 |
| Sirmaur | Himachal Pradesh | 32 | 0.85 | 0.72 | 1.00 | 0.88 | 0.76 | 1.02 |
| Shimla | Himachal Pradesh | 33 | 1.01 | 0.88 | 1.14 | 0.54 | 0.46 | 0.63 |
| Kinnaur | Himachal Pradesh | 34 | 0.85 | 0.54 | 1.23 | 1.17 | 0.83 | 1.61 |
| Gurdaspur | Punjab | 35 | 0.54 | 0.49 | 0.60 | 0.52 | 0.47 | 0.57 |
| Kapurthala | Punjab | 36 | 0.70 | 0.60 | 0.80 | 0.59 | 0.51 | 0.68 |
| Jalandhar | Punjab | 37 | 0.58 | 0.53 | 0.64 | 0.67 | 0.61 | 0.73 |
| Hoshiarpur | Punjab | 38 | 0.50 | 0.44 | 0.57 | 0.50 | 0.45 | 0.56 |
| Shahid Bhagat Singh Nagar | Punjab | 39 | 0.66 | 0.55 | 0.78 | 0.48 | 0.40 | 0.58 |
| Fatehgarh Sahib | Punjab | 40 | 0.66 | 0.54 | 0.78 | 0.91 | 0.79 | 1.05 |
| Ludhiana | Punjab | 41 | 0.70 | 0.65 | 0.76 | 0.72 | 0.67 | 0.77 |
| Moga | Punjab | 42 | 0.34 | 0.28 | 0.41 | 0.43 | 0.37 | 0.50 |
| Firozpur | Punjab | 43 | 0.60 | 0.54 | 0.67 | 0.50 | 0.45 | 0.56 |
| Muktsar | Punjab | 44 | 0.65 | 0.56 | 0.75 | 0.46 | 0.39 | 0.53 |
| Faridkot | Punjab | 45 | 0.59 | 0.48 | 0.71 | 0.44 | 0.36 | 0.53 |
| Bathinda | Punjab | 46 | 0.53 | 0.47 | 0.61 | 0.61 | 0.54 | 0.68 |
| Mansa | Punjab | 47 | 0.57 | 0.48 | 0.67 | 0.45 | 0.38 | 0.54 |
| Patiala | Punjab | 48 | 0.62 | 0.56 | 0.69 | 0.62 | 0.56 | 0.68 |
| Amritsar | Punjab | 49 | 0.61 | 0.55 | 0.66 | 0.58 | 0.53 | 0.63 |
| Tarn Taran | Punjab | 50 | 0.43 | 0.36 | 0.50 | 0.33 | 0.28 | 0.39 |
| Rupnagar | Punjab | 51 | 0.60 | 0.50 | 0.71 | 0.47 | 0.39 | 0.56 |
| Sahibzada Ajit Singh Nagar | Punjab | 52 | 0.96 | 0.85 | 1.08 | 1.20 | 1.09 | 1.32 |
| Sangrur | Punjab | 53 | 0.55 | 0.49 | 0.62 | 0.43 | 0.38 | 0.49 |
| Barnala | Punjab | 54 | 0.61 | 0.50 | 0.73 | 0.47 | 0.38 | 0.57 |
| Chandigarh | Chandigarh | 55 | 0.98 | 0.87 | 1.09 | 0.88 | 0.79 | 0.98 |
| Uttarkashi | Uttarakhand | 56 | 0.40 | 0.29 | 0.53 | 0.42 | 0.32 | 0.54 |
| Chamoli | Uttarakhand | 57 | 0.17 | 0.11 | 0.24 | 0.41 | 0.32 | 0.52 |
| Rudraprayag | Uttarakhand | 58 | 0.24 | 0.16 | 0.36 | 0.20 | 0.13 | 0.29 |
| Tehri Garhwal | Uttarakhand | 59 | 0.22 | 0.16 | 0.28 | 0.29 | 0.23 | 0.36 |
| Dehradun | Uttarakhand | 60 | 1.26 | 1.16 | 1.36 | 1.99 | 1.88 | 2.10 |
| Garhwal | Uttarakhand | 61 | 0.35 | 0.28 | 0.43 | 0.49 | 0.41 | 0.57 |
| Pithoragarh | Uttarakhand | 62 | 0.37 | 0.28 | 0.47 | 0.68 | 0.57 | 0.80 |
| Bageshwar | Uttarakhand | 63 | 0.66 | 0.51 | 0.84 | 0.27 | 0.18 | 0.38 |
| Almora | Uttarakhand | 64 | 0.15 | 0.10 | 0.20 | 0.23 | 0.18 | 0.30 |
| Champawat | Uttarakhand | 65 | 0.31 | 0.21 | 0.44 | 0.41 | 0.30 | 0.55 |
| Nainital | Uttarakhand | 66 | 1.31 | 1.18 | 1.44 | 1.67 | 1.54 | 1.81 |
| Udham Singh Nagar | Uttarakhand | 67 | 1.60 | 1.50 | 1.72 | 1.67 | 1.57 | 1.77 |
| Hardwar | Uttarakhand | 68 | 1.18 | 1.09 | 1.27 | 1.48 | 1.39 | 1.58 |
| Panchkula | Haryana | 69 | 1.52 | 1.34 | 1.70 | 1.93 | 1.75 | 2.13 |
| Ambala | Haryana | 70 | 1.60 | 1.47 | 1.73 | 1.96 | 1.83 | 2.10 |
| Yamunanagar | Haryana | 71 | 2.05 | 1.91 | 2.20 | 1.96 | 1.83 | 2.09 |
| Kurukshetra | Haryana | 72 | 2.41 | 2.24 | 2.59 | 2.39 | 2.23 | 2.56 |
| Kaithal | Haryana | 73 | 1.67 | 1.53 | 1.81 | 1.23 | 1.12 | 1.34 |
| Karnal | Haryana | 74 | 1.70 | 1.59 | 1.82 | 1.89 | 1.78 | 2.01 |
| Panipat | Haryana | 75 | 2.30 | 2.14 | 2.45 | 2.89 | 2.74 | 3.06 |
| Sonipat | Haryana | 76 | 1.82 | 1.69 | 1.95 | 2.13 | 2.01 | 2.25 |
| Jind | Haryana | 77 | 1.01 | 0.92 | 1.11 | 1.13 | 1.04 | 1.23 |
| Fatehabad | Haryana | 78 | 0.90 | 0.79 | 1.01 | 1.11 | 1.00 | 1.22 |
| Sirsa | Haryana | 79 | 1.32 | 1.21 | 1.43 | 1.34 | 1.24 | 1.44 |
| Hisar | Haryana | 80 | 1.49 | 1.39 | 1.60 | 1.91 | 1.81 | 2.02 |
| Bhiwani | Haryana | 81 | 1.36 | 1.26 | 1.46 | 1.65 | 1.55 | 1.75 |
| Rohtak | Haryana | 82 | 2.44 | 2.27 | 2.61 | 1.83 | 1.70 | 1.97 |
| Jhajjar | Haryana | 83 | 1.67 | 1.53 | 1.83 | 1.89 | 1.75 | 2.04 |
| Mahendragarh | Haryana | 84 | 1.07 | 0.95 | 1.19 | 0.94 | 0.84 | 1.05 |
| Rewari | Haryana | 85 | 2.02 | 1.86 | 2.19 | 1.51 | 1.38 | 1.65 |
| Gurgaon | Haryana | 86 | 2.43 | 2.29 | 2.57 | 2.48 | 2.35 | 2.62 |
| Mewat | Haryana | 87 | 1.05 | 0.94 | 1.16 | 1.06 | 0.96 | 1.16 |
| Faridabad | Haryana | 88 | 2.01 | 1.89 | 2.13 | 1.99 | 1.88 | 2.10 |
| Palwal | Haryana | 89 | 1.15 | 1.04 | 1.27 | 2.06 | 1.92 | 2.20 |
| North West | NCT of Delhi | 90 | 1.92 | 1.84 | 2.00 | 1.39 | 1.33 | 1.45 |
| North | NCT of Delhi | 91 | 3.81 | 3.58 | 4.04 | 5.70 | 5.45 | 5.96 |
| North East | NCT of Delhi | 92 | 1.37 | 1.29 | 1.46 | 1.92 | 1.83 | 2.01 |
| East | NCT of Delhi | 93 | 3.93 | 3.77 | 4.10 | 2.01 | 1.90 | 2.12 |
| New Delhi | NCT of Delhi | 94 | 3.50 | 2.98 | 4.07 | 2.90 | 2.47 | 3.38 |
| Central | NCT of Delhi | 95 | 2.55 | 2.33 | 2.79 | 2.97 | 2.75 | 3.20 |
| West | NCT of Delhi | 96 | 0.94 | 0.88 | 1.01 | 1.77 | 1.69 | 1.85 |
| South West | NCT of Delhi | 97 | 2.23 | 2.12 | 2.34 | 1.80 | 1.71 | 1.89 |
| South | NCT of Delhi | 98 | 0.74 | 0.69 | 0.80 | 2.51 | 2.42 | 2.61 |
| Ganganagar | Rajasthan | 99 | 2.30 | 2.18 | 2.42 | 2.28 | 2.17 | 2.39 |
| Hanumangarh | Rajasthan | 100 | 1.88 | 1.77 | 2.00 | 2.09 | 1.98 | 2.20 |
| Bikaner | Rajasthan | 101 | 1.30 | 1.22 | 1.39 | 1.38 | 1.30 | 1.46 |
| Churu | Rajasthan | 102 | 1.13 | 1.05 | 1.22 | 1.16 | 1.09 | 1.24 |
| Jhunjhunun | Rajasthan | 103 | 1.11 | 1.03 | 1.19 | 1.12 | 1.05 | 1.20 |
| Alwar | Rajasthan | 104 | 2.00 | 1.92 | 2.08 | 2.15 | 2.08 | 2.23 |
| Bharatpur | Rajasthan | 105 | 2.00 | 1.90 | 2.10 | 2.07 | 1.98 | 2.17 |
| Dhaulpur | Rajasthan | 106 | 1.97 | 1.83 | 2.12 | 1.88 | 1.75 | 2.01 |
| Karauli | Rajasthan | 107 | 1.41 | 1.30 | 1.52 | 1.81 | 1.70 | 1.93 |
| Sawai Madhopur | Rajasthan | 108 | 1.72 | 1.60 | 1.85 | 1.72 | 1.61 | 1.84 |
| Dausa | Rajasthan | 109 | 1.05 | 0.96 | 1.14 | 1.27 | 1.18 | 1.36 |
| Jaipur | Rajasthan | 110 | 1.51 | 1.45 | 1.56 | 1.88 | 1.82 | 1.93 |
| Sikar | Rajasthan | 111 | 1.20 | 1.13 | 1.27 | 1.42 | 1.35 | 1.49 |
| Nagaur | Rajasthan | 112 | 1.02 | 0.96 | 1.08 | 1.14 | 1.08 | 1.20 |
| Jodhpur | Rajasthan | 113 | 1.38 | 1.31 | 1.45 | 1.50 | 1.44 | 1.57 |
| Jaisalmer | Rajasthan | 114 | 1.24 | 1.09 | 1.40 | 1.15 | 1.02 | 1.29 |
| Barmer | Rajasthan | 115 | 1.49 | 1.41 | 1.58 | 1.15 | 1.09 | 1.22 |
| Jalor | Rajasthan | 116 | 1.08 | 0.99 | 1.16 | 1.06 | 0.99 | 1.14 |
| Sirohi | Rajasthan | 117 | 1.79 | 1.65 | 1.94 | 1.95 | 1.82 | 2.09 |
| Pali | Rajasthan | 118 | 1.86 | 1.76 | 1.97 | 1.89 | 1.80 | 1.99 |
| Ajmer | Rajasthan | 119 | 2.28 | 2.18 | 2.39 | 2.55 | 2.45 | 2.65 |
| Tonk | Rajasthan | 120 | 1.45 | 1.34 | 1.56 | 1.42 | 1.32 | 1.52 |
| Bundi | Rajasthan | 121 | 2.12 | 1.97 | 2.28 | 1.98 | 1.85 | 2.12 |
| Bhilwara | Rajasthan | 122 | 1.75 | 1.66 | 1.84 | 2.51 | 2.41 | 2.61 |
| Rajsamand | Rajasthan | 123 | 1.74 | 1.61 | 1.88 | 1.66 | 1.55 | 1.78 |
| Dungarpur | Rajasthan | 124 | 1.18 | 1.08 | 1.28 | 1.20 | 1.11 | 1.29 |
| Banswara | Rajasthan | 125 | 0.92 | 0.84 | 1.00 | 1.11 | 1.04 | 1.19 |
| Chittaurgarh | Rajasthan | 126 | 1.96 | 1.84 | 2.08 | 1.95 | 1.84 | 2.06 |
| Kota | Rajasthan | 127 | 2.13 | 2.02 | 2.25 | 2.33 | 2.22 | 2.45 |
| Baran | Rajasthan | 128 | 2.35 | 2.20 | 2.50 | 1.89 | 1.76 | 2.01 |
| Jhalawar | Rajasthan | 129 | 2.57 | 2.42 | 2.72 | 2.27 | 2.15 | 2.40 |
| Udaipur | Rajasthan | 130 | 1.28 | 1.21 | 1.36 | 1.91 | 1.83 | 1.99 |
| Pratapgarh | Rajasthan | 131 | 2.31 | 2.14 | 2.49 | 2.79 | 2.62 | 2.97 |
| Saharanpur | Uttar Pradesh | 132 | 0.72 | 0.67 | 0.77 | 0.70 | 0.65 | 0.75 |
| Muzaffarnagar | Uttar Pradesh | 133 | 0.58 | 0.54 | 0.62 | 0.76 | 0.71 | 0.80 |
| Bijnor | Uttar Pradesh | 134 | 0.79 | 0.74 | 0.84 | 1.03 | 0.97 | 1.08 |
| Moradabad | Uttar Pradesh | 135 | 1.09 | 1.03 | 1.14 | 0.86 | 0.82 | 0.90 |
| Rampur | Uttar Pradesh | 136 | 0.59 | 0.53 | 0.65 | 1.07 | 1.00 | 1.14 |
| Jyotiba Phule Nagar | Uttar Pradesh | 137 | 0.75 | 0.68 | 0.82 | 0.02 | 0.02 | 0.04 |
| Meerut | Uttar Pradesh | 138 | 0.75 | 0.70 | 0.80 | 1.82 | 1.75 | 1.90 |
| Baghpat | Uttar Pradesh | 139 | 1.03 | 0.93 | 1.13 | 1.30 | 1.20 | 1.40 |
| Ghaziabad | Uttar Pradesh | 140 | 0.55 | 0.51 | 0.59 | 0.94 | 0.89 | 0.99 |
| Gautam Buddha Nagar | Uttar Pradesh | 141 | 1.57 | 1.47 | 1.69 | 1.60 | 1.50 | 1.70 |
| Bulandshahr | Uttar Pradesh | 142 | 1.18 | 1.12 | 1.25 | 1.00 | 0.94 | 1.05 |
| Aligarh | Uttar Pradesh | 143 | 1.43 | 1.36 | 1.50 | 1.83 | 1.76 | 1.91 |
| Mahamaya Nagar | Uttar Pradesh | 144 | 0.97 | 0.88 | 1.06 | 1.11 | 1.03 | 1.20 |
| Mathura | Uttar Pradesh | 145 | 1.47 | 1.38 | 1.55 | 1.57 | 1.49 | 1.65 |
| Agra | Uttar Pradesh | 146 | 0.77 | 0.72 | 0.81 | 1.01 | 0.97 | 1.06 |
| Firozabad | Uttar Pradesh | 147 | 1.21 | 1.13 | 1.28 | 2.01 | 1.92 | 2.10 |
| Mainpuri | Uttar Pradesh | 148 | 1.01 | 0.93 | 1.09 | 1.02 | 0.94 | 1.09 |
| Budaun | Uttar Pradesh | 149 | 0.55 | 0.51 | 0.60 | 0.93 | 0.88 | 0.98 |
| Bareilly | Uttar Pradesh | 150 | 0.87 | 0.82 | 0.92 | 1.38 | 1.32 | 1.44 |
| Pilibhit | Uttar Pradesh | 151 | 1.09 | 1.01 | 1.17 | 1.71 | 1.62 | 1.81 |
| Shahjahanpur | Uttar Pradesh | 152 | 0.96 | 0.89 | 1.02 | 0.70 | 0.65 | 0.75 |
| Kheri | Uttar Pradesh | 153 | 0.94 | 0.89 | 1.00 | 0.56 | 0.53 | 0.60 |
| Sitapur | Uttar Pradesh | 154 | 0.88 | 0.83 | 0.93 | 0.59 | 0.56 | 0.63 |
| Hardoi | Uttar Pradesh | 155 | 0.50 | 0.47 | 0.54 | 0.51 | 0.47 | 0.55 |
| Unnao | Uttar Pradesh | 156 | 0.95 | 0.89 | 1.01 | 0.77 | 0.72 | 0.82 |
| Lucknow | Uttar Pradesh | 157 | 2.00 | 1.92 | 2.07 | 1.28 | 1.23 | 1.34 |
| Rae Bareli | Uttar Pradesh | 158 | 0.77 | 0.72 | 0.83 | 0.63 | 0.59 | 0.68 |
| Farrukhabad | Uttar Pradesh | 159 | 0.65 | 0.58 | 0.71 | 0.74 | 0.68 | 0.80 |
| Kannauj | Uttar Pradesh | 160 | 0.99 | 0.91 | 1.08 | 1.80 | 1.69 | 1.91 |
| Etawah | Uttar Pradesh | 161 | 0.63 | 0.56 | 0.70 | 0.65 | 0.59 | 0.72 |
| Auraiya | Uttar Pradesh | 162 | 1.11 | 1.01 | 1.21 | 1.36 | 1.26 | 1.47 |
| Kanpur Dehat | Uttar Pradesh | 163 | 1.38 | 1.28 | 1.48 | 1.02 | 0.95 | 1.10 |
| Kanpur Nagar | Uttar Pradesh | 164 | 1.12 | 1.06 | 1.17 | 1.31 | 1.25 | 1.36 |
| Jalaun | Uttar Pradesh | 165 | 0.72 | 0.65 | 0.80 | 0.61 | 0.55 | 0.67 |
| Jhansi | Uttar Pradesh | 166 | 1.11 | 1.03 | 1.20 | 0.83 | 0.76 | 0.90 |
| Lalitpur | Uttar Pradesh | 167 | 0.31 | 0.26 | 0.37 | 0.48 | 0.42 | 0.55 |
| Hamirpur | Uttar Pradesh | 168 | 0.94 | 0.84 | 1.04 | 1.04 | 0.95 | 1.15 |
| Mahoba | Uttar Pradesh | 169 | 0.78 | 0.68 | 0.89 | 0.67 | 0.59 | 0.77 |
| Banda | Uttar Pradesh | 170 | 0.91 | 0.83 | 0.99 | 1.18 | 1.10 | 1.27 |
| Chitrakoot | Uttar Pradesh | 171 | 0.71 | 0.62 | 0.81 | 0.58 | 0.51 | 0.66 |
| Fatehpur | Uttar Pradesh | 172 | 0.76 | 0.70 | 0.82 | 0.63 | 0.59 | 0.69 |
| Pratapgarh | Uttar Pradesh | 173 | 0.98 | 0.92 | 1.04 | 0.92 | 0.87 | 0.97 |
| Kaushambi | Uttar Pradesh | 174 | 0.76 | 0.69 | 0.84 | 0.66 | 0.60 | 0.73 |
| Allahabad | Uttar Pradesh | 175 | 0.95 | 0.91 | 1.00 | 0.91 | 0.87 | 0.95 |
| Bara Banki | Uttar Pradesh | 176 | 0.73 | 0.68 | 0.78 | 1.21 | 1.15 | 1.27 |
| Faizabad | Uttar Pradesh | 177 | 0.99 | 0.92 | 1.06 | 1.16 | 1.09 | 1.23 |
| Ambedkar Nagar | Uttar Pradesh | 178 | 0.60 | 0.54 | 0.65 | 0.67 | 0.62 | 0.72 |
| Sultanpur | Uttar Pradesh | 179 | 0.66 | 0.62 | 0.71 | 0.81 | 0.77 | 0.86 |
| Bahraich | Uttar Pradesh | 180 | 0.86 | 0.81 | 0.92 | 1.60 | 1.53 | 1.67 |
| Shrawasti | Uttar Pradesh | 181 | 0.83 | 0.73 | 0.93 | 1.06 | 0.97 | 1.17 |
| Balrampur | Uttar Pradesh | 182 | 0.52 | 0.47 | 0.58 | 0.74 | 0.68 | 0.80 |
| Gonda | Uttar Pradesh | 183 | 0.56 | 0.52 | 0.61 | 0.67 | 0.62 | 0.71 |
| Siddharth Nagar | Uttar Pradesh | 184 | 0.42 | 0.38 | 0.47 | 0.54 | 0.50 | 0.59 |
| Basti | Uttar Pradesh | 185 | 0.43 | 0.39 | 0.48 | 0.87 | 0.82 | 0.93 |
| Sant Kabir Nagar | Uttar Pradesh | 186 | 0.53 | 0.47 | 0.59 | 0.66 | 0.60 | 0.73 |
| Maharajganj | Uttar Pradesh | 187 | 0.50 | 0.45 | 0.54 | 0.33 | 0.29 | 0.36 |
| Gorakhpur | Uttar Pradesh | 188 | 0.64 | 0.60 | 0.68 | 0.94 | 0.89 | 0.98 |
| Kushinagar | Uttar Pradesh | 189 | 0.45 | 0.41 | 0.48 | 0.56 | 0.52 | 0.60 |
| Deoria | Uttar Pradesh | 190 | 0.09 | 0.08 | 0.11 | 0.10 | 0.08 | 0.11 |
| Azamgarh | Uttar Pradesh | 191 | 0.50 | 0.47 | 0.54 | 0.68 | 0.64 | 0.72 |
| Mau | Uttar Pradesh | 192 | 0.73 | 0.67 | 0.80 | 0.83 | 0.77 | 0.89 |
| Ballia | Uttar Pradesh | 193 | 0.37 | 0.33 | 0.40 | 0.50 | 0.46 | 0.54 |
| Jaunpur | Uttar Pradesh | 194 | 0.35 | 0.32 | 0.38 | 0.50 | 0.47 | 0.53 |
| Ghazipur | Uttar Pradesh | 195 | 0.61 | 0.56 | 0.65 | 0.61 | 0.57 | 0.65 |
| Chandauli | Uttar Pradesh | 196 | 0.39 | 0.34 | 0.44 | 0.41 | 0.36 | 0.46 |
| Varanasi | Uttar Pradesh | 197 | 0.95 | 0.89 | 1.01 | 0.74 | 0.70 | 0.79 |
| Sant Ravi Das Nagar(bhadohi) | Uttar Pradesh | 198 | 0.40 | 0.34 | 0.45 | 0.52 | 0.46 | 0.58 |
| Mirzapur | Uttar Pradesh | 199 | 0.38 | 0.34 | 0.42 | 0.34 | 0.31 | 0.38 |
| Sonbhadra | Uttar Pradesh | 200 | 0.48 | 0.42 | 0.54 | 0.67 | 0.61 | 0.73 |
| Etah | Uttar Pradesh | 201 | 0.75 | 0.68 | 0.82 | 0.53 | 0.48 | 0.59 |
| Kansiram Nagar | Uttar Pradesh | 202 | 1.72 | 1.59 | 1.84 | 1.24 | 1.14 | 1.33 |
| Pashchim Champaran | Bihar | 203 | 0.49 | 0.45 | 0.52 | 0.42 | 0.39 | 0.45 |
| Purba Champaran | Bihar | 204 | 0.42 | 0.38 | 0.45 | 0.51 | 0.48 | 0.54 |
| Sheohar | Bihar | 205 | 0.45 | 0.36 | 0.55 | 0.52 | 0.44 | 0.62 |
| Sitamarhi | Bihar | 206 | 0.46 | 0.42 | 0.50 | 0.68 | 0.64 | 0.73 |
| Madhubani | Bihar | 207 | 0.35 | 0.32 | 0.38 | 0.22 | 0.20 | 0.25 |
| Supaul | Bihar | 208 | 0.35 | 0.31 | 0.40 | 0.35 | 0.32 | 0.40 |
| Araria | Bihar | 209 | 0.43 | 0.39 | 0.48 | 0.44 | 0.40 | 0.48 |
| Kishanganj | Bihar | 210 | 0.48 | 0.42 | 0.54 | 0.43 | 0.38 | 0.48 |
| Purnia | Bihar | 211 | 0.38 | 0.34 | 0.41 | 0.55 | 0.51 | 0.59 |
| Katihar | Bihar | 212 | 0.47 | 0.43 | 0.52 | 0.43 | 0.40 | 0.47 |
| Madhepura | Bihar | 213 | 0.34 | 0.30 | 0.39 | 0.38 | 0.34 | 0.43 |
| Saharsa | Bihar | 214 | 0.27 | 0.23 | 0.31 | 0.30 | 0.26 | 0.34 |
| Darbhanga | Bihar | 215 | 0.43 | 0.40 | 0.47 | 0.27 | 0.25 | 0.30 |
| Muzaffarpur | Bihar | 216 | 0.52 | 0.48 | 0.55 | 0.67 | 0.64 | 0.71 |
| Gopalganj | Bihar | 217 | 0.35 | 0.31 | 0.39 | 0.49 | 0.44 | 0.53 |
| Siwan | Bihar | 218 | 0.30 | 0.27 | 0.33 | 0.34 | 0.31 | 0.37 |
| Saran (chhapra) | Bihar | 219 | 0.58 | 0.53 | 0.62 | 0.59 | 0.55 | 0.63 |
| Vaishali | Bihar | 220 | 0.55 | 0.51 | 0.60 | 0.56 | 0.52 | 0.60 |
| Samastipur | Bihar | 221 | 0.32 | 0.29 | 0.35 | 0.36 | 0.33 | 0.39 |
| Begusarai | Bihar | 222 | 0.29 | 0.26 | 0.33 | 0.37 | 0.34 | 0.41 |
| Khagaria | Bihar | 223 | 0.38 | 0.33 | 0.43 | 0.58 | 0.52 | 0.64 |
| Bhagalpur | Bihar | 224 | 0.74 | 0.68 | 0.79 | 0.73 | 0.68 | 0.78 |
| Banka | Bihar | 225 | 0.38 | 0.33 | 0.43 | 0.34 | 0.30 | 0.38 |
| Munger | Bihar | 226 | 0.59 | 0.52 | 0.66 | 0.66 | 0.60 | 0.74 |
| Lakhisarai | Bihar | 227 | 0.47 | 0.40 | 0.54 | 0.60 | 0.52 | 0.68 |
| Sheikhpura | Bihar | 228 | 0.55 | 0.45 | 0.65 | 0.63 | 0.54 | 0.74 |
| Nalanda | Bihar | 229 | 0.73 | 0.68 | 0.79 | 0.73 | 0.68 | 0.78 |
| Patna | Bihar | 230 | 0.86 | 0.82 | 0.91 | 0.91 | 0.87 | 0.95 |
| Bhojpur | Bihar | 231 | 0.62 | 0.57 | 0.68 | 0.58 | 0.53 | 0.62 |
| Buxar | Bihar | 232 | 0.54 | 0.48 | 0.60 | 0.48 | 0.43 | 0.53 |
| Kaimur (bhabua) | Bihar | 233 | 0.43 | 0.37 | 0.48 | 0.40 | 0.36 | 0.45 |
| Rohtas | Bihar | 234 | 0.37 | 0.34 | 0.41 | 0.38 | 0.35 | 0.42 |
| Aurangabad | Bihar | 235 | 0.29 | 0.25 | 0.33 | 0.45 | 0.41 | 0.49 |
| Gaya | Bihar | 236 | 0.46 | 0.43 | 0.50 | 0.71 | 0.68 | 0.76 |
| Nawada | Bihar | 237 | 0.45 | 0.40 | 0.50 | 0.49 | 0.44 | 0.53 |
| Jamui | Bihar | 238 | 0.38 | 0.33 | 0.43 | 0.34 | 0.30 | 0.39 |
| Jehanabad | Bihar | 239 | 0.36 | 0.30 | 0.43 | 0.45 | 0.39 | 0.51 |
| Arwal | Bihar | 240 | 0.38 | 0.30 | 0.46 | 0.48 | 0.40 | 0.56 |
| North | Sikkim | 241 | 0.72 | 0.36 | 1.21 | 0.87 | 0.50 | 1.42 |
| West | Sikkim | 242 | 0.43 | 0.26 | 0.63 | 0.68 | 0.49 | 0.94 |
| South | Sikkim | 243 | 0.82 | 0.58 | 1.10 | 0.82 | 0.60 | 1.08 |
| East | Sikkim | 244 | 1.01 | 0.81 | 1.24 | 0.95 | 0.77 | 1.15 |
| Tawang | Arunanchal Pradesh | 245 | 0.14 | 0.04 | 0.35 | 0.28 | 0.11 | 0.59 |
| West Kameng | Arunanchal Pradesh | 246 | 0.43 | 0.23 | 0.69 | 0.36 | 0.20 | 0.60 |
| East Kameng | Arunanchal Pradesh | 247 | 0.33 | 0.17 | 0.55 | 0.60 | 0.39 | 0.90 |
| Papum Pare | Arunanchal Pradesh | 248 | 2.06 | 1.70 | 2.45 | 2.31 | 1.96 | 2.69 |
| Upper Subansiri | Arunanchal Pradesh | 249 | 0.63 | 0.38 | 0.95 | 0.35 | 0.19 | 0.58 |
| West Siang | Arunanchal Pradesh | 250 | 0.75 | 0.50 | 1.04 | 0.95 | 0.69 | 1.27 |
| East Siang | Arunanchal Pradesh | 251 | 1.27 | 0.92 | 1.68 | 0.77 | 0.53 | 1.08 |
| Upper Siang | Arunanchal Pradesh | 252 | 0.42 | 0.18 | 0.79 | 0.60 | 0.31 | 1.06 |
| Changlang | Arunanchal Pradesh | 253 | 0.37 | 0.23 | 0.55 | 0.33 | 0.21 | 0.50 |
| Tirap | Arunanchal Pradesh | 254 | 0.32 | 0.18 | 0.51 | 0.21 | 0.11 | 0.35 |
| Lower Subansiri | Arunanchal Pradesh | 255 | 0.31 | 0.17 | 0.52 | 0.36 | 0.21 | 0.58 |
| Kurung Kumey | Arunanchal Pradesh | 256 | 0.29 | 0.15 | 0.49 | 0.16 | 0.08 | 0.30 |
| Dibang Valley | Arunanchal Pradesh | 257 | 0.98 | 0.27 | 2.29 | 0.40 | 0.09 | 1.13 |
| Lower Dibang Valley | Arunanchal Pradesh | 258 | 0.49 | 0.27 | 0.80 | 0.59 | 0.34 | 0.93 |
| Lohit | Arunanchal Pradesh | 259 | 0.64 | 0.43 | 0.88 | 0.43 | 0.28 | 0.62 |
| Anjaw | Arunanchal Pradesh | 260 | 0.28 | 0.08 | 0.64 | 0.54 | 0.22 | 1.11 |
| Mon | Nagaland | 261 | 0.10 | 0.05 | 0.17 | 0.10 | 0.05 | 0.17 |
| Mokokchung | Nagaland | 262 | 0.11 | 0.06 | 0.19 | 0.09 | 0.05 | 0.16 |
| Zunheboto | Nagaland | 263 | 0.09 | 0.04 | 0.16 | 0.07 | 0.03 | 0.14 |
| Wokha | Nagaland | 264 | 0.12 | 0.06 | 0.22 | 0.08 | 0.04 | 0.15 |
| Dimapur | Nagaland | 265 | 0.19 | 0.12 | 0.27 | 0.11 | 0.07 | 0.18 |
| Phek | Nagaland | 266 | 0.06 | 0.02 | 0.11 | 0.07 | 0.03 | 0.13 |
| Tuensang | Nagaland | 267 | 0.05 | 0.02 | 0.10 | 0.07 | 0.03 | 0.14 |
| Longleng | Nagaland | 268 | 0.12 | 0.04 | 0.26 | 0.14 | 0.06 | 0.31 |
| Kiphire | Nagaland | 269 | 0.06 | 0.02 | 0.15 | 0.06 | 0.02 | 0.15 |
| Kohima | Nagaland | 270 | 0.09 | 0.05 | 0.15 | 0.11 | 0.06 | 0.18 |
| Peren | Nagaland | 271 | 0.11 | 0.05 | 0.21 | 0.13 | 0.06 | 0.24 |
| Senapati | Manipur | 272 | 0.08 | 0.04 | 0.11 | 0.05 | 0.03 | 0.09 |
| Tamenglong | Manipur | 273 | 0.11 | 0.05 | 0.20 | 0.11 | 0.05 | 0.19 |
| Churachandpur | Manipur | 274 | 0.15 | 0.09 | 0.23 | 0.16 | 0.10 | 0.23 |
| Bishnupur | Manipur | 275 | 0.41 | 0.28 | 0.56 | 0.22 | 0.14 | 0.32 |
| Thoubal | Manipur | 276 | 0.47 | 0.37 | 0.59 | 0.34 | 0.26 | 0.44 |
| Imphal West | Manipur | 277 | 0.53 | 0.42 | 0.64 | 0.50 | 0.41 | 0.60 |
| Imphal East | Manipur | 278 | 0.30 | 0.22 | 0.39 | 0.27 | 0.20 | 0.35 |
| Ukhrul | Manipur | 279 | 0.05 | 0.02 | 0.10 | 0.08 | 0.04 | 0.16 |
| Chandel | Manipur | 280 | 0.12 | 0.06 | 0.22 | 0.13 | 0.07 | 0.23 |
| Mamit | Mizoram | 281 | 0.75 | 0.48 | 1.08 | 0.44 | 0.27 | 0.69 |
| Kolasib | Mizoram | 282 | 0.88 | 0.57 | 1.25 | 0.43 | 0.25 | 0.68 |
| Aizawl | Mizoram | 283 | 0.52 | 0.41 | 0.65 | 0.44 | 0.34 | 0.55 |
| Champhai | Mizoram | 284 | 0.68 | 0.45 | 0.95 | 0.46 | 0.30 | 0.67 |
| Serchhip | Mizoram | 285 | 0.33 | 0.15 | 0.58 | 0.49 | 0.28 | 0.80 |
| Lunglei | Mizoram | 286 | 0.40 | 0.25 | 0.57 | 0.22 | 0.13 | 0.35 |
| Lawangtlai | Mizoram | 287 | 0.34 | 0.19 | 0.55 | 0.21 | 0.11 | 0.36 |
| Saiha | Mizoram | 288 | 0.28 | 0.11 | 0.54 | 0.29 | 0.13 | 0.56 |
| West Tripura | Tripura | 289 | 0.77 | 0.70 | 0.85 | 0.42 | 0.37 | 0.47 |
| South Tripura | Tripura | 290 | 0.91 | 0.79 | 1.02 | 0.65 | 0.56 | 0.74 |
| Dhalai | Tripura | 291 | 0.48 | 0.36 | 0.61 | 0.49 | 0.38 | 0.62 |
| North Tripura | Tripura | 292 | 0.81 | 0.69 | 0.93 | 0.86 | 0.75 | 0.98 |
| West Garo Hills | Meghalaya | 293 | 0.45 | 0.37 | 0.55 | 0.66 | 0.56 | 0.77 |
| East Garo Hills | Meghalaya | 294 | 0.44 | 0.33 | 0.58 | 0.50 | 0.39 | 0.64 |
| South Garo Hills | Meghalaya | 295 | 0.54 | 0.35 | 0.77 | 0.49 | 0.33 | 0.70 |
| West Khasi Hills | Meghalaya | 296 | 0.66 | 0.53 | 0.81 | 0.56 | 0.45 | 0.69 |
| Ri Bhoi | Meghalaya | 297 | 1.05 | 0.84 | 1.28 | 0.71 | 0.56 | 0.89 |
| East Khasi Hills | Meghalaya | 298 | 0.63 | 0.53 | 0.72 | 0.70 | 0.62 | 0.80 |
| Jaintia Hills | Meghalaya | 299 | 0.82 | 0.67 | 0.99 | 0.65 | 0.53 | 0.79 |
| Kokrajhar | Assam | 300 | 1.47 | 1.33 | 1.61 | 1.06 | 0.96 | 1.18 |
| Dhubri | Assam | 301 | 4.68 | 4.50 | 4.85 | 1.25 | 1.17 | 1.33 |
| Goalpara | Assam | 302 | 2.03 | 1.87 | 2.19 | 1.80 | 1.67 | 1.94 |
| Barpeta | Assam | 303 | 4.39 | 4.21 | 4.57 | 1.10 | 1.02 | 1.18 |
| Marigaon | Assam | 304 | 4.26 | 4.03 | 4.49 | 1.54 | 1.42 | 1.67 |
| Nagaon | Assam | 305 | 3.20 | 3.08 | 3.32 | 1.32 | 1.25 | 1.39 |
| Sonitpur | Assam | 306 | 1.66 | 1.55 | 1.76 | 1.41 | 1.33 | 1.50 |
| Lakhimpur | Assam | 307 | 2.96 | 2.78 | 3.15 | 1.03 | 0.93 | 1.13 |
| Dhemaji | Assam | 308 | 2.02 | 1.84 | 2.22 | 0.79 | 0.69 | 0.91 |
| Tinsukia | Assam | 309 | 1.64 | 1.52 | 1.77 | 1.01 | 0.92 | 1.10 |
| Dibrugarh | Assam | 310 | 2.05 | 1.91 | 2.19 | 0.80 | 0.73 | 0.89 |
| Sivasagar | Assam | 311 | 2.61 | 2.44 | 2.78 | 0.88 | 0.79 | 0.97 |
| Jorhat | Assam | 312 | 3.60 | 3.40 | 3.80 | 1.00 | 0.91 | 1.10 |
| Golaghat | Assam | 313 | 1.99 | 1.84 | 2.15 | 0.50 | 0.44 | 0.58 |
| Karbi Anglong | Assam | 314 | 0.99 | 0.88 | 1.11 | 0.29 | 0.24 | 0.35 |
| Dima Hasao | Assam | 315 | 0.68 | 0.50 | 0.88 | 0.39 | 0.28 | 0.54 |
| Cachar | Assam | 316 | 1.92 | 1.80 | 2.04 | 1.57 | 1.48 | 1.67 |
| Karimganj | Assam | 317 | 1.46 | 1.34 | 1.58 | 1.05 | 0.96 | 1.14 |
| Hailakandi | Assam | 318 | 3.24 | 3.00 | 3.49 | 1.24 | 1.11 | 1.39 |
| Bongaigaon | Assam | 319 | 3.39 | 3.15 | 3.63 | 1.03 | 0.92 | 1.16 |
| Chirang | Assam | 320 | 2.02 | 1.80 | 2.25 | 0.91 | 0.78 | 1.05 |
| Kamrup | Assam | 321 | 3.33 | 3.17 | 3.50 | 2.18 | 2.06 | 2.30 |
| Kamrup Metropolitan | Assam | 322 | 4.31 | 4.11 | 4.52 | 2.14 | 2.01 | 2.28 |
| Nalbari | Assam | 323 | 2.64 | 2.44 | 2.85 | 1.29 | 1.16 | 1.42 |
| Baksa | Assam | 324 | 1.48 | 1.34 | 1.62 | 0.59 | 0.51 | 0.67 |
| Darrang | Assam | 325 | 4.09 | 3.86 | 4.33 | 2.19 | 2.03 | 2.35 |
| Udalguri | Assam | 326 | 1.17 | 1.05 | 1.31 | 0.83 | 0.74 | 0.94 |
| Darjiling | West Bengal | 327 | 1.72 | 1.61 | 1.83 | 0.68 | 0.61 | 0.74 |
| Jalpaiguri | West Bengal | 328 | 1.81 | 1.74 | 1.89 | 1.62 | 1.55 | 1.69 |
| Koch Bihar | West Bengal | 329 | 1.33 | 1.25 | 1.41 | 1.30 | 1.23 | 1.37 |
| Uttar Dinajpur | West Bengal | 330 | 0.35 | 0.31 | 0.39 | 0.74 | 0.69 | 0.79 |
| Dakshin Dinajpur | West Bengal | 331 | 0.87 | 0.79 | 0.95 | 0.81 | 0.74 | 0.88 |
| Maldah | West Bengal | 332 | 1.25 | 1.19 | 1.32 | 1.28 | 1.23 | 1.35 |
| Murshidabad | West Bengal | 333 | 1.71 | 1.65 | 1.76 | 1.25 | 1.21 | 1.30 |
| Birbhum | West Bengal | 334 | 0.60 | 0.55 | 0.65 | 0.51 | 0.47 | 0.55 |
| Barddhaman | West Bengal | 335 | 1.16 | 1.11 | 1.20 | 0.84 | 0.80 | 0.87 |
| Nadia | West Bengal | 336 | 1.73 | 1.66 | 1.79 | 1.23 | 1.18 | 1.28 |
| North 24 Parganas | West Bengal | 337 | 2.34 | 2.29 | 2.40 | 1.10 | 1.06 | 1.13 |
| Hugli | West Bengal | 338 | 0.92 | 0.87 | 0.97 | 0.79 | 0.75 | 0.83 |
| Bankura | West Bengal | 339 | 0.49 | 0.45 | 0.53 | 2.32 | 2.24 | 2.41 |
| Puruliya | West Bengal | 340 | 0.45 | 0.41 | 0.50 | 0.35 | 0.31 | 0.38 |
| Haora | West Bengal | 341 | 1.19 | 1.13 | 1.25 | 0.94 | 0.89 | 0.99 |
| Kolkata | West Bengal | 342 | 1.55 | 1.48 | 1.61 | 2.20 | 2.13 | 2.28 |
| South 24 Parganas | West Bengal | 343 | 1.56 | 1.51 | 1.61 | 0.95 | 0.91 | 0.98 |
| Pashchim Medinipur | West Bengal | 344 | 0.98 | 0.93 | 1.02 | 0.67 | 0.64 | 0.71 |
| Purba Medinipur | West Bengal | 345 | 1.29 | 1.23 | 1.35 | 0.99 | 0.95 | 1.04 |
| Garhwa | Jharkhand | 346 | 1.36 | 1.25 | 1.48 | 0.80 | 0.72 | 0.88 |
| Chatra | Jharkhand | 347 | 0.62 | 0.54 | 0.71 | 0.43 | 0.37 | 0.50 |
| Kodarma | Jharkhand | 348 | 0.44 | 0.36 | 0.53 | 0.76 | 0.67 | 0.87 |
| Giridih | Jharkhand | 349 | 0.48 | 0.44 | 0.53 | 0.63 | 0.58 | 0.68 |
| Deoghar | Jharkhand | 350 | 1.50 | 1.39 | 1.61 | 0.83 | 0.76 | 0.91 |
| Godda | Jharkhand | 351 | 0.68 | 0.60 | 0.76 | 1.01 | 0.93 | 1.11 |
| Sahibganj | Jharkhand | 352 | 1.01 | 0.91 | 1.11 | 0.67 | 0.60 | 0.75 |
| Pakur | Jharkhand | 353 | 0.45 | 0.38 | 0.53 | 0.76 | 0.67 | 0.85 |
| Dhanbad | Jharkhand | 354 | 0.82 | 0.76 | 0.88 | 0.59 | 0.55 | 0.64 |
| Bokaro | Jharkhand | 355 | 1.05 | 0.97 | 1.13 | 0.58 | 0.53 | 0.63 |
| Lohardaga | Jharkhand | 356 | 0.71 | 0.58 | 0.85 | 0.84 | 0.72 | 0.98 |
| Purbi Singhbhum | Jharkhand | 357 | 0.77 | 0.71 | 0.83 | 0.57 | 0.53 | 0.63 |
| Palamu | Jharkhand | 358 | 0.67 | 0.61 | 0.74 | 0.75 | 0.69 | 0.81 |
| Latehar | Jharkhand | 359 | 0.74 | 0.63 | 0.85 | 0.65 | 0.57 | 0.75 |
| Hazaribagh | Jharkhand | 360 | 0.83 | 0.76 | 0.91 | 0.69 | 0.63 | 0.75 |
| Ramgarh | Jharkhand | 361 | 0.75 | 0.65 | 0.85 | 0.48 | 0.41 | 0.56 |
| Dumka | Jharkhand | 362 | 0.51 | 0.45 | 0.58 | 0.42 | 0.37 | 0.48 |
| Jamtara | Jharkhand | 363 | 0.51 | 0.43 | 0.60 | 0.50 | 0.43 | 0.59 |
| Ranchi | Jharkhand | 364 | 0.62 | 0.57 | 0.67 | 0.55 | 0.50 | 0.59 |
| Khunti | Jharkhand | 365 | 0.50 | 0.40 | 0.61 | 0.42 | 0.34 | 0.51 |
| Gumla | Jharkhand | 366 | 0.59 | 0.51 | 0.68 | 0.67 | 0.59 | 0.75 |
| Simdega | Jharkhand | 367 | 0.34 | 0.27 | 0.43 | 0.48 | 0.40 | 0.57 |
| Pashchimi Singhbhum | Jharkhand | 368 | 0.39 | 0.34 | 0.45 | 0.24 | 0.21 | 0.29 |
| Saraikela-kharsawan | Jharkhand | 369 | 0.42 | 0.36 | 0.49 | 0.33 | 0.28 | 0.39 |
| Bargarh | Odisha | 370 | 1.11 | 1.01 | 1.21 | 1.30 | 1.21 | 1.40 |
| Jharsuguda | Odisha | 371 | 2.50 | 2.27 | 2.74 | 1.29 | 1.14 | 1.45 |
| Sambalpur | Odisha | 372 | 2.47 | 2.30 | 2.64 | 1.45 | 1.34 | 1.58 |
| Debagarh | Odisha | 373 | 2.12 | 1.84 | 2.42 | 4.00 | 3.64 | 4.38 |
| Sundargarh | Odisha | 374 | 1.55 | 1.45 | 1.65 | 1.35 | 1.27 | 1.44 |
| Kendujhar | Odisha | 375 | 1.39 | 1.30 | 1.49 | 1.60 | 1.51 | 1.70 |
| Mayurbhanj | Odisha | 376 | 1.91 | 1.82 | 2.01 | 1.94 | 1.85 | 2.03 |
| Baleshwar | Odisha | 377 | 2.31 | 2.20 | 2.42 | 1.03 | 0.96 | 1.10 |
| Bhadrak | Odisha | 378 | 1.93 | 1.80 | 2.06 | 1.33 | 1.24 | 1.43 |
| Kendrapara | Odisha | 379 | 3.18 | 3.02 | 3.35 | 2.80 | 2.66 | 2.94 |
| Jagatsinghapur | Odisha | 380 | 3.07 | 2.89 | 3.26 | 2.20 | 2.06 | 2.34 |
| Cuttack | Odisha | 381 | 1.63 | 1.54 | 1.72 | 1.53 | 1.46 | 1.62 |
| Jajapur | Odisha | 382 | 2.55 | 2.41 | 2.68 | 1.34 | 1.26 | 1.43 |
| Dhenkanal | Odisha | 383 | 3.06 | 2.88 | 3.24 | 1.70 | 1.58 | 1.83 |
| Anugul | Odisha | 384 | 3.27 | 3.09 | 3.45 | 2.93 | 2.77 | 3.09 |
| Nayagarh | Odisha | 385 | 1.80 | 1.65 | 1.96 | 1.79 | 1.65 | 1.93 |
| Khordha | Odisha | 386 | 1.86 | 1.76 | 1.97 | 1.92 | 1.82 | 2.02 |
| Puri | Odisha | 387 | 3.01 | 2.86 | 3.16 | 1.04 | 0.96 | 1.12 |
| Ganjam | Odisha | 388 | 1.57 | 1.49 | 1.64 | 1.65 | 1.58 | 1.72 |
| Gajapati | Odisha | 389 | 1.48 | 1.31 | 1.66 | 1.00 | 0.88 | 1.14 |
| Kandhamal | Odisha | 390 | 1.54 | 1.38 | 1.70 | 2.21 | 2.04 | 2.39 |
| Bauda | Odisha | 391 | 1.57 | 1.37 | 1.79 | 0.88 | 0.75 | 1.04 |
| Subarnapur | Odisha | 392 | 0.96 | 0.82 | 1.10 | 1.09 | 0.96 | 1.24 |
| Balangir | Odisha | 393 | 1.93 | 1.81 | 2.05 | 0.87 | 0.80 | 0.95 |
| Nuapada | Odisha | 394 | 1.27 | 1.11 | 1.43 | 1.00 | 0.88 | 1.14 |
| Kalahandi | Odisha | 395 | 1.73 | 1.62 | 1.85 | 1.36 | 1.27 | 1.46 |
| Rayagada | Odisha | 396 | 1.79 | 1.64 | 1.94 | 0.98 | 0.88 | 1.09 |
| Nabarangapur | Odisha | 397 | 1.77 | 1.64 | 1.91 | 0.75 | 0.67 | 0.83 |
| Koraput | Odisha | 398 | 1.50 | 1.39 | 1.62 | 1.39 | 1.29 | 1.50 |
| Malkangiri | Odisha | 399 | 2.33 | 2.11 | 2.55 | 2.50 | 2.30 | 2.71 |
| Koriya | Chhattisgarh | 400 | 1.36 | 1.21 | 1.52 | 1.38 | 1.24 | 1.53 |
| Surguja | Chhattisgarh | 401 | 1.13 | 1.06 | 1.21 | 1.08 | 1.02 | 1.15 |
| Jashpur | Chhattisgarh | 402 | 0.94 | 0.83 | 1.06 | 0.88 | 0.78 | 0.98 |
| Raigarh | Chhattisgarh | 403 | 1.00 | 0.92 | 1.09 | 1.01 | 0.93 | 1.09 |
| Korba | Chhattisgarh | 404 | 0.77 | 0.69 | 0.86 | 0.73 | 0.66 | 0.81 |
| Janjgir-champa | Chhattisgarh | 405 | 0.87 | 0.79 | 0.95 | 1.15 | 1.07 | 1.24 |
| Bilaspur | Chhattisgarh | 406 | 1.11 | 1.04 | 1.18 | 0.99 | 0.93 | 1.06 |
| Kabeerdham | Chhattisgarh | 407 | 0.82 | 0.72 | 0.93 | 0.78 | 0.69 | 0.88 |
| Rajnandgaon | Chhattisgarh | 408 | 0.56 | 0.50 | 0.63 | 0.66 | 0.60 | 0.72 |
| Durg | Chhattisgarh | 409 | 0.89 | 0.84 | 0.95 | 0.82 | 0.78 | 0.87 |
| Raipur | Chhattisgarh | 410 | 1.01 | 0.96 | 1.07 | 1.04 | 0.99 | 1.09 |
| Mahasamund | Chhattisgarh | 411 | 0.61 | 0.53 | 0.69 | 0.58 | 0.51 | 0.66 |
| Dhamtari | Chhattisgarh | 412 | 0.82 | 0.71 | 0.93 | 0.55 | 0.48 | 0.64 |
| Uttar Bastar Kanker | Chhattisgarh | 413 | 0.74 | 0.64 | 0.85 | 0.49 | 0.42 | 0.58 |
| Bastar | Chhattisgarh | 414 | 0.58 | 0.52 | 0.65 | 0.62 | 0.56 | 0.69 |
| Narayanpur | Chhattisgarh | 415 | 1.15 | 0.87 | 1.47 | 0.78 | 0.58 | 1.04 |
| Dakshin Bastar Dantewada | Chhattisgarh | 416 | 0.51 | 0.41 | 0.61 | 0.59 | 0.49 | 0.70 |
| Bijapur | Chhattisgarh | 417 | 0.49 | 0.36 | 0.64 | 0.55 | 0.42 | 0.71 |
| Sheopur | Madhya Pradesh | 418 | 0.71 | 0.60 | 0.82 | 0.67 | 0.58 | 0.77 |
| Morena | Madhya Pradesh | 419 | 0.72 | 0.66 | 0.79 | 1.03 | 0.96 | 1.11 |
| Bhind | Madhya Pradesh | 420 | 0.72 | 0.65 | 0.79 | 0.56 | 0.51 | 0.62 |
| Gwalior | Madhya Pradesh | 421 | 2.32 | 2.20 | 2.44 | 1.98 | 1.88 | 2.09 |
| Datia | Madhya Pradesh | 422 | 0.79 | 0.68 | 0.91 | 0.89 | 0.79 | 1.00 |
| Shivpuri | Madhya Pradesh | 423 | 1.01 | 0.93 | 1.10 | 1.12 | 1.04 | 1.20 |
| Tikamgarh | Madhya Pradesh | 424 | 1.11 | 1.01 | 1.21 | 0.92 | 0.85 | 1.01 |
| Chhatarpur | Madhya Pradesh | 425 | 1.04 | 0.96 | 1.13 | 1.15 | 1.07 | 1.23 |
| Panna | Madhya Pradesh | 426 | 1.36 | 1.23 | 1.49 | 1.19 | 1.08 | 1.30 |
| Sagar | Madhya Pradesh | 427 | 1.19 | 1.11 | 1.27 | 1.52 | 1.44 | 1.60 |
| Damoh | Madhya Pradesh | 428 | 1.32 | 1.21 | 1.44 | 1.05 | 0.96 | 1.15 |
| Satna | Madhya Pradesh | 429 | 0.95 | 0.88 | 1.03 | 0.80 | 0.74 | 0.86 |
| Rewa | Madhya Pradesh | 430 | 1.06 | 0.99 | 1.13 | 1.17 | 1.10 | 1.24 |
| Umaria | Madhya Pradesh | 431 | 0.77 | 0.66 | 0.90 | 0.64 | 0.55 | 0.75 |
| Neemuch | Madhya Pradesh | 432 | 0.67 | 0.57 | 0.77 | 1.27 | 1.15 | 1.39 |
| Mandsaur | Madhya Pradesh | 433 | 0.75 | 0.67 | 0.83 | 0.90 | 0.82 | 0.98 |
| Ratlam | Madhya Pradesh | 434 | 1.29 | 1.19 | 1.39 | 1.35 | 1.26 | 1.45 |
| Ujjain | Madhya Pradesh | 435 | 1.08 | 1.00 | 1.16 | 1.10 | 1.03 | 1.18 |
| Shajapur | Madhya Pradesh | 436 | 1.05 | 0.96 | 1.15 | 1.03 | 0.95 | 1.12 |
| Dewas | Madhya Pradesh | 437 | 1.30 | 1.20 | 1.40 | 0.98 | 0.91 | 1.07 |
| Dhar | Madhya Pradesh | 438 | 0.81 | 0.74 | 0.88 | 1.43 | 1.35 | 1.51 |
| Indore | Madhya Pradesh | 439 | 1.67 | 1.59 | 1.75 | 0.36 | 0.33 | 0.40 |
| West Nimar | Madhya Pradesh | 440 | 0.87 | 0.80 | 0.94 | 1.01 | 0.94 | 1.08 |
| Barwani | Madhya Pradesh | 441 | 0.72 | 0.64 | 0.80 | 0.87 | 0.80 | 0.95 |
| Rajgarh | Madhya Pradesh | 442 | 1.02 | 0.93 | 1.11 | 1.58 | 1.48 | 1.68 |
| Vidisha | Madhya Pradesh | 443 | 1.31 | 1.20 | 1.41 | 1.17 | 1.08 | 1.27 |
| Bhopal | Madhya Pradesh | 444 | 1.78 | 1.68 | 1.88 | 2.60 | 2.49 | 2.71 |
| Sehore | Madhya Pradesh | 445 | 1.15 | 1.05 | 1.25 | 1.17 | 1.08 | 1.27 |
| Raisen | Madhya Pradesh | 446 | 1.24 | 1.14 | 1.35 | 1.34 | 1.25 | 1.45 |
| Betul | Madhya Pradesh | 447 | 0.93 | 0.84 | 1.01 | 0.65 | 0.59 | 0.72 |
| Harda | Madhya Pradesh | 448 | 1.10 | 0.96 | 1.26 | 1.85 | 1.68 | 2.04 |
| Hoshangabad | Madhya Pradesh | 449 | 1.35 | 1.24 | 1.47 | 1.27 | 1.17 | 1.37 |
| Katni | Madhya Pradesh | 450 | 0.82 | 0.74 | 0.91 | 0.92 | 0.84 | 1.01 |
| Jabalpur | Madhya Pradesh | 451 | 1.65 | 1.56 | 1.74 | 1.69 | 1.61 | 1.78 |
| Narsimhapur | Madhya Pradesh | 452 | 0.86 | 0.77 | 0.96 | 1.35 | 1.24 | 1.46 |
| Dindori | Madhya Pradesh | 453 | 1.06 | 0.93 | 1.20 | 1.33 | 1.20 | 1.47 |
| Mandla | Madhya Pradesh | 454 | 0.79 | 0.70 | 0.89 | 0.90 | 0.81 | 0.99 |
| Chhindwara | Madhya Pradesh | 455 | 0.65 | 0.59 | 0.71 | 0.53 | 0.49 | 0.59 |
| Seoni | Madhya Pradesh | 456 | 0.97 | 0.88 | 1.07 | 1.08 | 1.00 | 1.18 |
| Balaghat | Madhya Pradesh | 457 | 1.22 | 1.13 | 1.31 | 0.85 | 0.79 | 0.93 |
| Guna | Madhya Pradesh | 458 | 1.61 | 1.48 | 1.74 | 0.76 | 0.69 | 0.84 |
| Ashoknagar | Madhya Pradesh | 459 | 1.42 | 1.28 | 1.57 | 1.33 | 1.21 | 1.46 |
| Shahdol | Madhya Pradesh | 460 | 1.50 | 1.37 | 1.63 | 1.38 | 1.27 | 1.50 |
| Anuppur | Madhya Pradesh | 461 | 1.40 | 1.26 | 1.55 | 1.34 | 1.21 | 1.47 |
| Sidhi | Madhya Pradesh | 462 | 0.70 | 0.62 | 0.79 | 0.90 | 0.81 | 0.99 |
| Singrauli | Madhya Pradesh | 463 | 1.00 | 0.90 | 1.11 | 1.08 | 0.99 | 1.18 |
| Jhabua | Madhya Pradesh | 464 | 0.53 | 0.45 | 0.61 | 0.94 | 0.85 | 1.04 |
| Alirajpur | Madhya Pradesh | 465 | 0.84 | 0.73 | 0.95 | 0.59 | 0.50 | 0.68 |
| East Nimar | Madhya Pradesh | 466 | 1.02 | 0.93 | 1.12 | 1.06 | 0.97 | 1.15 |
| Burhanpur | Madhya Pradesh | 467 | 0.78 | 0.67 | 0.89 | 1.18 | 1.06 | 1.31 |
| Kachchh | Gujarat | 468 | 0.49 | 0.44 | 0.54 | 0.44 | 0.40 | 0.49 |
| Banas Kantha | Gujarat | 469 | 0.38 | 0.34 | 0.42 | 0.20 | 0.17 | 0.22 |
| Patan | Gujarat | 470 | 0.49 | 0.42 | 0.56 | 0.26 | 0.22 | 0.31 |
| Mahesana | Gujarat | 471 | 0.27 | 0.23 | 0.31 | 0.30 | 0.26 | 0.34 |
| Sabar Kantha | Gujarat | 472 | 0.44 | 0.39 | 0.49 | 0.35 | 0.32 | 0.39 |
| Gandhinagar | Gujarat | 473 | 0.44 | 0.38 | 0.51 | 0.37 | 0.32 | 0.42 |
| Ahmadabad | Gujarat | 474 | 0.77 | 0.73 | 0.81 | 0.62 | 0.59 | 0.65 |
| Surendranagar | Gujarat | 475 | 0.23 | 0.19 | 0.27 | 0.19 | 0.16 | 0.23 |
| Rajkot | Gujarat | 476 | 0.48 | 0.44 | 0.52 | 0.46 | 0.42 | 0.49 |
| Jamnagar | Gujarat | 477 | 0.32 | 0.28 | 0.37 | 0.27 | 0.23 | 0.30 |
| Porbandar | Gujarat | 478 | 0.31 | 0.23 | 0.39 | 0.29 | 0.22 | 0.36 |
| Junagadh | Gujarat | 479 | 0.55 | 0.50 | 0.60 | 0.29 | 0.26 | 0.32 |
| Amreli | Gujarat | 480 | 0.49 | 0.43 | 0.56 | 0.35 | 0.31 | 0.40 |
| Bhavnagar | Gujarat | 481 | 0.39 | 0.35 | 0.43 | 0.27 | 0.24 | 0.30 |
| Anand | Gujarat | 482 | 0.32 | 0.28 | 0.37 | 0.27 | 0.24 | 0.31 |
| Kheda | Gujarat | 483 | 0.37 | 0.33 | 0.41 | 0.31 | 0.27 | 0.34 |
| Panch Mahals | Gujarat | 484 | 0.31 | 0.27 | 0.35 | 0.29 | 0.26 | 0.33 |
| Dohad | Gujarat | 485 | 0.32 | 0.28 | 0.37 | 0.31 | 0.27 | 0.35 |
| Vadodara | Gujarat | 486 | 0.51 | 0.47 | 0.54 | 0.31 | 0.29 | 0.34 |
| Narmada | Gujarat | 487 | 0.33 | 0.26 | 0.42 | 0.27 | 0.21 | 0.35 |
| Bharuch | Gujarat | 488 | 0.40 | 0.35 | 0.46 | 0.34 | 0.29 | 0.39 |
| The Dangs | Gujarat | 489 | 0.19 | 0.11 | 0.28 | 0.07 | 0.04 | 0.13 |
| Navsari | Gujarat | 490 | 0.15 | 0.12 | 0.19 | 0.12 | 0.09 | 0.15 |
| Valsad | Gujarat | 491 | 0.16 | 0.13 | 0.20 | 0.16 | 0.13 | 0.19 |
| Surat | Gujarat | 492 | 0.44 | 0.40 | 0.47 | 0.47 | 0.44 | 0.50 |
| Tapi | Gujarat | 493 | 0.12 | 0.09 | 0.17 | 0.08 | 0.05 | 0.11 |
| Diu | Daman & Diu | 494 | 0.32 | 0.14 | 0.59 | 0.11 | 0.03 | 0.26 |
| Daman | Daman & Diu | 495 | 0.35 | 0.21 | 0.52 | 0.75 | 0.57 | 0.97 |
| Dadra & Nagar Haveli | Dadara & Nagar Havelli | 496 | 0.28 | 0.20 | 0.37 | 0.42 | 0.33 | 0.52 |
| Nandurbar | Maharashtra | 497 | 0.33 | 0.29 | 0.38 | 0.55 | 0.50 | 0.61 |
| Dhule | Maharashtra | 498 | 0.78 | 0.72 | 0.86 | 0.95 | 0.88 | 1.02 |
| Jalgaon | Maharashtra | 499 | 0.66 | 0.61 | 0.70 | 0.88 | 0.84 | 0.93 |
| Buldana | Maharashtra | 500 | 0.97 | 0.91 | 1.05 | 1.04 | 0.97 | 1.10 |
| Akola | Maharashtra | 501 | 1.32 | 1.22 | 1.42 | 1.18 | 1.10 | 1.27 |
| Washim | Maharashtra | 502 | 1.28 | 1.16 | 1.39 | 1.26 | 1.16 | 1.37 |
| Amravati | Maharashtra | 503 | 1.41 | 1.33 | 1.48 | 1.43 | 1.35 | 1.50 |
| Wardha | Maharashtra | 504 | 1.23 | 1.12 | 1.34 | 1.08 | 0.99 | 1.18 |
| Nagpur | Maharashtra | 505 | 0.94 | 0.89 | 0.99 | 1.03 | 0.99 | 1.08 |
| Bhandara | Maharashtra | 506 | 0.80 | 0.71 | 0.89 | 0.68 | 0.60 | 0.76 |
| Gondiya | Maharashtra | 507 | 0.49 | 0.42 | 0.56 | 0.76 | 0.68 | 0.84 |
| Garhchiroli | Maharashtra | 508 | 0.57 | 0.50 | 0.66 | 0.45 | 0.39 | 0.52 |
| Chandrapur | Maharashtra | 509 | 0.97 | 0.90 | 1.05 | 1.11 | 1.04 | 1.19 |
| Yavatmal | Maharashtra | 510 | 0.88 | 0.82 | 0.95 | 1.16 | 1.10 | 1.23 |
| Nanded | Maharashtra | 511 | 0.74 | 0.69 | 0.80 | 0.73 | 0.69 | 0.78 |
| Hingoli | Maharashtra | 512 | 0.64 | 0.56 | 0.72 | 0.94 | 0.85 | 1.04 |
| Parbhani | Maharashtra | 513 | 0.76 | 0.69 | 0.84 | 0.84 | 0.77 | 0.91 |
| Jalna | Maharashtra | 514 | 1.26 | 1.17 | 1.35 | 1.61 | 1.52 | 1.70 |
| Aurangabad | Maharashtra | 515 | 0.96 | 0.90 | 1.02 | 1.27 | 1.21 | 1.33 |
| Nashik | Maharashtra | 516 | 0.82 | 0.77 | 0.86 | 0.94 | 0.90 | 0.98 |
| Thane | Maharashtra | 517 | 0.86 | 0.83 | 0.90 | 1.26 | 1.22 | 1.29 |
| Mumbai Suburban | Maharashtra | 518 | 1.71 | 1.66 | 1.76 | 0.33 | 0.31 | 0.35 |
| Mumbai | Maharashtra | 519 | 0.79 | 0.73 | 0.85 | 6.09 | 5.94 | 6.25 |
| Raigarh | Maharashtra | 520 | 0.34 | 0.30 | 0.38 | 0.42 | 0.38 | 0.46 |
| Pune | Maharashtra | 521 | 1.16 | 1.12 | 1.20 | 1.64 | 1.59 | 1.68 |
| Ahmadnagar | Maharashtra | 522 | 1.20 | 1.15 | 1.26 | 1.36 | 1.30 | 1.42 |
| Bid | Maharashtra | 523 | 1.02 | 0.95 | 1.09 | 1.35 | 1.27 | 1.42 |
| Latur | Maharashtra | 524 | 0.62 | 0.57 | 0.68 | 0.90 | 0.84 | 0.96 |
| Osmanabad | Maharashtra | 525 | 0.74 | 0.67 | 0.82 | 0.85 | 0.77 | 0.92 |
| Solapur | Maharashtra | 526 | 1.00 | 0.94 | 1.05 | 1.01 | 0.96 | 1.06 |
| Satara | Maharashtra | 527 | 0.91 | 0.85 | 0.97 | 0.89 | 0.84 | 0.95 |
| Ratnagiri | Maharashtra | 528 | 0.34 | 0.30 | 0.40 | 0.32 | 0.28 | 0.37 |
| Sindhudurg | Maharashtra | 529 | 0.38 | 0.31 | 0.46 | 0.45 | 0.38 | 0.53 |
| Kolhapur | Maharashtra | 530 | 0.69 | 0.65 | 0.74 | 0.76 | 0.72 | 0.81 |
| Sangli | Maharashtra | 531 | 0.83 | 0.77 | 0.89 | 0.82 | 0.76 | 0.87 |
| Adilabad | Andhra Pradesh | 532 | 0.64 | 0.58 | 0.69 | 0.74 | 0.69 | 0.80 |
| Nizamabad | Andhra Pradesh | 533 | 1.03 | 0.96 | 1.10 | 1.17 | 1.10 | 1.24 |
| Karimnagar | Andhra Pradesh | 534 | 1.78 | 1.71 | 1.86 | 1.75 | 1.68 | 1.83 |
| Medak | Andhra Pradesh | 535 | 1.33 | 1.25 | 1.40 | 1.23 | 1.16 | 1.29 |
| Hyderabad | Andhra Pradesh | 536 | 2.07 | 1.99 | 2.15 | 2.31 | 2.23 | 2.40 |
| Rangareddy | Andhra Pradesh | 537 | 3.64 | 3.54 | 3.73 | 3.79 | 3.70 | 3.88 |
| Mahbubnagar | Andhra Pradesh | 538 | 0.83 | 0.78 | 0.88 | 0.98 | 0.93 | 1.03 |
| Nalgonda | Andhra Pradesh | 539 | 1.22 | 1.15 | 1.29 | 1.25 | 1.19 | 1.31 |
| Warangal | Andhra Pradesh | 540 | 1.46 | 1.39 | 1.53 | 2.90 | 2.81 | 3.00 |
| Khammam | Andhra Pradesh | 541 | 1.59 | 1.50 | 1.67 | 1.44 | 1.37 | 1.52 |
| Srikakulam | Andhra Pradesh | 542 | 0.56 | 0.51 | 0.61 | 0.67 | 0.62 | 0.73 |
| Vizianagaram | Andhra Pradesh | 543 | 0.89 | 0.83 | 0.97 | 1.15 | 1.08 | 1.23 |
| Visakhapatnam | Andhra Pradesh | 544 | 1.16 | 1.10 | 1.22 | 1.34 | 1.28 | 1.40 |
| East Godavari | Andhra Pradesh | 545 | 1.15 | 1.10 | 1.21 | 1.70 | 1.65 | 1.76 |
| West Godavari | Andhra Pradesh | 546 | 1.18 | 1.12 | 1.25 | 0.69 | 0.65 | 0.74 |
| Krishna | Andhra Pradesh | 547 | 1.78 | 1.71 | 1.86 | 2.50 | 2.43 | 2.58 |
| Guntur | Andhra Pradesh | 548 | 1.49 | 1.43 | 1.55 | 2.06 | 1.99 | 2.12 |
| Prakasam | Andhra Pradesh | 549 | 1.37 | 1.30 | 1.45 | 1.32 | 1.26 | 1.39 |
| Sri Potti Sriramulu Nellore | Andhra Pradesh | 550 | 1.07 | 1.00 | 1.14 | 1.14 | 1.07 | 1.20 |
| Y.s.r. | Andhra Pradesh | 551 | 0.76 | 0.70 | 0.82 | 1.08 | 1.02 | 1.15 |
| Kurnool | Andhra Pradesh | 552 | 1.39 | 1.33 | 1.46 | 1.22 | 1.16 | 1.28 |
| Anantapur | Andhra Pradesh | 553 | 0.93 | 0.88 | 0.99 | 0.93 | 0.88 | 0.98 |
| Chittoor | Andhra Pradesh | 554 | 0.71 | 0.66 | 0.75 | 1.19 | 1.14 | 1.25 |
| Belgaum | Karnataka | 555 | 0.42 | 0.38 | 0.45 | 0.51 | 0.48 | 0.55 |
| Bagalkot | Karnataka | 556 | 0.44 | 0.39 | 0.50 | 0.47 | 0.42 | 0.52 |
| Bijapur | Karnataka | 557 | 0.50 | 0.45 | 0.56 | 0.52 | 0.47 | 0.57 |
| Bidar | Karnataka | 558 | 0.44 | 0.38 | 0.49 | 0.47 | 0.42 | 0.53 |
| Raichur | Karnataka | 559 | 0.68 | 0.62 | 0.75 | 0.73 | 0.67 | 0.80 |
| Koppal | Karnataka | 560 | 0.60 | 0.53 | 0.67 | 0.66 | 0.59 | 0.73 |
| Gadag | Karnataka | 561 | 0.31 | 0.25 | 0.37 | 0.36 | 0.31 | 0.43 |
| Dharwad | Karnataka | 562 | 0.39 | 0.34 | 0.44 | 0.53 | 0.48 | 0.59 |
| Uttara Kannada | Karnataka | 563 | 0.53 | 0.46 | 0.60 | 0.64 | 0.58 | 0.72 |
| Haveri | Karnataka | 564 | 0.48 | 0.42 | 0.54 | 0.60 | 0.54 | 0.66 |
| Bellary | Karnataka | 565 | 0.61 | 0.55 | 0.66 | 0.60 | 0.55 | 0.65 |
| Chitradurga | Karnataka | 566 | 0.81 | 0.74 | 0.89 | 1.07 | 0.99 | 1.16 |
| Davanagere | Karnataka | 567 | 0.64 | 0.58 | 0.71 | 0.82 | 0.76 | 0.89 |
| Shimoga | Karnataka | 568 | 1.09 | 1.00 | 1.18 | 1.28 | 1.19 | 1.37 |
| Udupi | Karnataka | 569 | 0.56 | 0.49 | 0.64 | 0.52 | 0.46 | 0.59 |
| Chikmagalur | Karnataka | 570 | 0.85 | 0.76 | 0.95 | 1.07 | 0.98 | 1.18 |
| Tumkur | Karnataka | 571 | 0.42 | 0.38 | 0.46 | 0.74 | 0.69 | 0.79 |
| Bangalore | Karnataka | 572 | 0.97 | 0.93 | 1.01 | 1.17 | 1.14 | 1.21 |
| Mandya | Karnataka | 573 | 0.88 | 0.80 | 0.96 | 0.93 | 0.86 | 1.01 |
| Hassan | Karnataka | 574 | 1.10 | 1.01 | 1.19 | 1.00 | 0.92 | 1.08 |
| Dakshina Kannada | Karnataka | 575 | 0.48 | 0.43 | 0.53 | 0.56 | 0.51 | 0.61 |
| Kodagu | Karnataka | 576 | 1.06 | 0.92 | 1.22 | 0.91 | 0.79 | 1.05 |
| Mysore | Karnataka | 577 | 0.69 | 0.64 | 0.75 | 0.90 | 0.84 | 0.95 |
| Chamrajnagar | Karnataka | 578 | 0.70 | 0.61 | 0.80 | 1.10 | 0.99 | 1.21 |
| Gulbarga | Karnataka | 579 | 0.66 | 0.61 | 0.72 | 0.57 | 0.52 | 0.62 |
| Yadgir | Karnataka | 580 | 0.68 | 0.60 | 0.77 | 0.55 | 0.48 | 0.62 |
| Kolar | Karnataka | 581 | 0.51 | 0.44 | 0.57 | 0.82 | 0.75 | 0.90 |
| Chikkaballapura | Karnataka | 582 | 0.73 | 0.65 | 0.82 | 0.60 | 0.54 | 0.68 |
| Bangalore Rural | Karnataka | 583 | 1.19 | 1.07 | 1.32 | 2.01 | 1.86 | 2.16 |
| Ramanagara | Karnataka | 584 | 1.08 | 0.97 | 1.20 | 1.55 | 1.43 | 1.67 |
| North Goa | Goa | 585 | 0.54 | 0.45 | 0.63 | 0.58 | 0.50 | 0.67 |
| South Goa | Goa | 586 | 0.46 | 0.37 | 0.56 | 0.47 | 0.39 | 0.57 |
| Kasaragod | Kerala | 588 | 0.96 | 0.87 | 1.06 | 0.92 | 0.83 | 1.00 |
| Kannur | Kerala | 589 | 0.66 | 0.61 | 0.72 | 0.80 | 0.74 | 0.85 |
| Wayanad | Kerala | 590 | 1.32 | 1.18 | 1.46 | 2.03 | 1.88 | 2.20 |
| Kozhikode | Kerala | 591 | 1.07 | 1.01 | 1.14 | 1.55 | 1.48 | 1.62 |
| Malappuram | Kerala | 592 | 0.87 | 0.82 | 0.92 | 0.96 | 0.91 | 1.01 |
| Palakkad | Kerala | 593 | 0.65 | 0.60 | 0.71 | 0.87 | 0.81 | 0.93 |
| Thrissur | Kerala | 594 | 0.96 | 0.89 | 1.02 | 0.68 | 0.64 | 0.73 |
| Ernakulam | Kerala | 595 | 1.00 | 0.94 | 1.06 | 1.70 | 1.62 | 1.77 |
| Idukki | Kerala | 596 | 1.33 | 1.21 | 1.45 | 1.51 | 1.39 | 1.63 |
| Kottayam | Kerala | 597 | 0.65 | 0.58 | 0.71 | 1.01 | 0.93 | 1.08 |
| Alappuzha | Kerala | 598 | 0.84 | 0.77 | 0.91 | 1.02 | 0.95 | 1.10 |
| Pathanamthitta | Kerala | 599 | 1.05 | 0.95 | 1.15 | 1.39 | 1.28 | 1.50 |
| Kollam | Kerala | 600 | 1.31 | 1.23 | 1.39 | 1.69 | 1.61 | 1.78 |
| Thiruvananthapuram | Kerala | 601 | 1.30 | 1.23 | 1.37 | 1.74 | 1.67 | 1.82 |
| Thiruvallur | Tamil Nadu | 602 | 0.11 | 0.09 | 0.13 | 0.27 | 0.24 | 0.30 |
| Chennai | Tamil Nadu | 603 | 0.42 | 0.39 | 0.45 | 0.53 | 0.50 | 0.57 |
| Kancheepuram | Tamil Nadu | 604 | 0.11 | 0.09 | 0.12 | 0.09 | 0.07 | 0.10 |
| Vellore | Tamil Nadu | 605 | 0.28 | 0.25 | 0.31 | 0.19 | 0.17 | 0.22 |
| Tiruvannamalai | Tamil Nadu | 606 | 0.24 | 0.20 | 0.27 | 0.24 | 0.21 | 0.27 |
| Viluppuram | Tamil Nadu | 607 | 0.41 | 0.38 | 0.45 | 0.31 | 0.28 | 0.35 |
| Salem | Tamil Nadu | 608 | 0.32 | 0.29 | 0.36 | 0.25 | 0.22 | 0.28 |
| Namakkal | Tamil Nadu | 609 | 0.25 | 0.21 | 0.29 | 0.31 | 0.26 | 0.35 |
| Erode | Tamil Nadu | 610 | 0.23 | 0.20 | 0.27 | 0.30 | 0.26 | 0.34 |
| The Nilgiris | Tamil Nadu | 611 | 0.40 | 0.32 | 0.48 | 0.36 | 0.29 | 0.43 |
| Dindigul | Tamil Nadu | 612 | 0.33 | 0.29 | 0.37 | 0.34 | 0.30 | 0.38 |
| Karur | Tamil Nadu | 613 | 0.27 | 0.21 | 0.32 | 0.33 | 0.28 | 0.39 |
| Tiruchirappalli | Tamil Nadu | 614 | 0.37 | 0.33 | 0.41 | 0.38 | 0.34 | 0.42 |
| Perambalur | Tamil Nadu | 615 | 0.36 | 0.27 | 0.45 | 0.53 | 0.43 | 0.63 |
| Ariyalur | Tamil Nadu | 616 | 0.30 | 0.23 | 0.37 | 0.48 | 0.40 | 0.57 |
| Cuddalore | Tamil Nadu | 617 | 0.38 | 0.34 | 0.42 | 0.45 | 0.41 | 0.49 |
| Nagappattinam | Tamil Nadu | 618 | 0.30 | 0.25 | 0.35 | 0.36 | 0.31 | 0.41 |
| Thiruvarur | Tamil Nadu | 619 | 0.45 | 0.39 | 0.52 | 0.28 | 0.24 | 0.34 |
| Thanjavur | Tamil Nadu | 620 | 0.29 | 0.25 | 0.33 | 0.44 | 0.40 | 0.49 |
| Pudukkottai | Tamil Nadu | 621 | 0.43 | 0.38 | 0.49 | 0.33 | 0.28 | 0.38 |
| Sivaganga | Tamil Nadu | 622 | 0.39 | 0.33 | 0.45 | 0.50 | 0.44 | 0.57 |
| Madurai | Tamil Nadu | 623 | 0.49 | 0.44 | 0.53 | 0.57 | 0.53 | 0.62 |
| Theni | Tamil Nadu | 624 | 0.37 | 0.31 | 0.43 | 0.75 | 0.67 | 0.83 |
| Virudunagar | Tamil Nadu | 625 | 0.24 | 0.20 | 0.28 | 0.41 | 0.36 | 0.46 |
| Ramanathapuram | Tamil Nadu | 626 | 0.44 | 0.38 | 0.51 | 0.44 | 0.38 | 0.50 |
| Thoothukkudi | Tamil Nadu | 627 | 0.41 | 0.36 | 0.47 | 0.44 | 0.39 | 0.49 |
| Tirunelveli | Tamil Nadu | 628 | 0.38 | 0.34 | 0.42 | 0.49 | 0.45 | 0.53 |
| Kanniyakumari | Tamil Nadu | 629 | 0.38 | 0.33 | 0.43 | 0.33 | 0.29 | 0.38 |
| Dharmapuri | Tamil Nadu | 630 | 0.27 | 0.22 | 0.32 | 0.36 | 0.31 | 0.41 |
| Krishnagiri | Tamil Nadu | 631 | 0.18 | 0.15 | 0.22 | 0.22 | 0.19 | 0.26 |
| Coimbatore | Tamil Nadu | 632 | 0.21 | 0.19 | 0.24 | 0.38 | 0.35 | 0.42 |
| Tiruppur | Tamil Nadu | 633 | 0.23 | 0.19 | 0.26 | 0.31 | 0.28 | 0.35 |
| Nicobar | Andaman & Nicobar Island | 638 | 2.95 | 1.96 | 4.12 | 2.85 | 1.99 | 3.97 |
| North & Middle Andaman | Andaman & Nicobar Island | 639 | 1.76 | 1.32 | 2.26 | 0.79 | 0.54 | 1.13 |
| South Andaman | Andaman & Nicobar Island | 640 | 0.94 | 0.72 | 1.19 | 1.51 | 1.26 | 1.80 |
